# Does Product Placement Change Television Viewers’ Social Behavior?

**DOI:** 10.1371/journal.pone.0138610

**Published:** 2015-09-23

**Authors:** Elizabeth Levy Paluck, Paul Lagunes, Donald P. Green, Lynn Vavreck, Limor Peer, Robin Gomila

**Affiliations:** 1 Department of Psychology and Woodrow Wilson School of Public and International Affairs, Princeton University, Peretsman Scully Hall, Princeton, New Jersey, United States of America; 2 School of International and Public Affairs, Columbia University, New York City, New York, United States of America; 3 Department of Political Science, Columbia University, New York City, New York, United States of America; 4 Department of Political Science and Communication Studies, University of California Los Angeles, Los Angeles, California, United States of America; 5 Institution for Social and Policy Studies, Yale University, New Haven, Connecticut, United States of America; 6 Department of Psychology, Peretsman Scully Hall, Princeton University, Princeton, New Jersey, United States of America; University of Tuebingen Medical School, GERMANY

## Abstract

To what extent are television viewers affected by the behaviors and decisions they see modeled by characters in television soap operas? Collaborating with scriptwriters for three prime-time nationally-broadcast Spanish-language telenovelas, we embedded scenes about topics such as drunk driving or saving money at randomly assigned periods during the broadcast season. Outcomes were measured unobtrusively by aggregate city- and nation-wide time series, such as the number of Hispanic motorists arrested daily for drunk driving or the number of accounts opened in banks located in Hispanic neighborhoods. Results indicate that while two of the treatment effects are statistically significant, none are substantively large or long-lasting. Actions that could be taken during the immediate viewing session, like online searching, and those that were relatively more integrated into the telenovela storyline, specifically reducing cholesterol, were briefly affected, but not behaviors requiring sustained efforts, like opening a bank account or registering to vote.

## Introduction

Commercial product placement, defined as “insertion of branded products or services into mass media content with the intent of influencing consumer attitude or behavior” [1], is widely believed to affect audiences’ purchasing behavior. Evidence suggesting the influence of product placement comes from four sources: (i) examples of placement’s effects on consumer demand for which there is no ready alternative explanation, such as the surge in the popularity of the Pottery Barn brand after it appeared in an episode of the television series *Friends* in the 1990s [2], (ii) the enormous fees that companies are willing to pay to have their products featured in films and television shows [3], (iii) observational studies that find a strong correlation between media exposure to products (e.g., cigarettes in feature-length films) and preferences for those products [4], and (iv) laboratory experiments conducted under controlled conditions that demonstrate how awareness and evaluations of products rise when they appear in a television program or film [5]. Noticeably absent from this list are field experiments showing that demand increases in the wake of randomly assigned, naturalistic exposure to product placements in mass media, although such studies may have been conducted by commercial entities and exist outside the public domain.

The theoretical mechanisms by which product placement affects consumer behavior are several. Among them Bandura’s social learning theory [6], which posits that individuals acquire new response tendencies through modeling and imitative behaviors, especially when products are associated with characters with whom viewers identify [7, 8]. Additionally, elaboration likelihood theories of cognitive processing contend that viewers are especially susceptible to the influence of products and messages that are unobtrusively woven into plotlines because these subtle forms of influence do not provoke counter-arguing, especially when viewers are engrossed by the story and its characters [9–11].

The evidence and theory behind product placement’s effects on consumer behavior raise the question of whether similar tactics could be used to achieve social goals such as promoting literacy or reducing domestic violence. Media producers also deploy this kind of “behavioral product placement,” often by partnering with nonprofit or governmental organizations that have experience with a social issue. For example, the Spanish-language media company Univision recently announced that their media programming would use their media products to increase Hispanic college readiness, health, financial literacy, and voter participation: “We are taking our efforts to inform and empower our community to the next level …I am confident we will have an even greater impact” [12]. Additionally, the Spanish-language media company Telemundo recently partnered with the US Census Bureau to create scenes of Hispanic characters participating in the Census within one of Telemundo’s soap operas, or *telenovelas*. “It’s the perfect vehicle for product placement” noted one Census representative, “[it’s] people placement” [13, 14].

Soap operas in particular have traditionally been vehicles for behavioral product placement; socially-minded soap opera production teams all over the globe have used the medium to feature behaviors and attitudes they wished to promote in the viewing population [15]. The soap opera format suits many of the theoretical conditions of successful behavioral influence as reviewed above: they are considered entertaining and immersive, and the fact that they are aired daily over long stretches of time allows viewers to become highly involved and often to identify with the characters. It is also fitting that soap operas are used for behavioral product placement, given their historical ties to commercial product placement—the very name “soap opera” originates with the sponsorship of soap companies, whose advertising teams believed that the daytime dramas were excellent vehicles for selling cleaning products [16].

Sometimes, akin to commercial product placements where a product is featured briefly in a few scenes, soap opera protagonists model a targeted behavior or make a statement about their position on a topic in just one or two scenes [2]. Other soap operas integrate the behavior or social issue into the central theme of the show. A growing body of field experimental evidence suggests that this latter strategy of thematically focusing on a message can have strong and enduring effects on outcomes like women’s independence [17, 18], interethnic cooperation,[19] and literacy [20]. However, no comparable field experiments have evaluated the effects of inserting character behaviors or decisions into a few episodes or scenes, analogous to brief commercial product placements that insert goods or services.

The small body of experimental research on both kinds of product placement to date has been confined to laboratory-like settings, measuring outcomes immediately after subjects viewed treatment and control episodes [14]. Observational research on the population-level effects of product placement broadcast is unable to isolate the causal impact of the product placement against alternative explanations, such as concurrent trends in the popularity of the behavior [21]. Embedding normatively positive behaviors or decisions into mass media, particularly into entertainment media, in principle represents a cost-effective means for ameliorating social problems. However, the lack of field-based research on the causal impact of product placement leaves open the question of whether a mass media product placement strategy could effect beneficial social outcomes. A research design providing a method to evaluate the causal impact of product placements on population-level behavioral outcomes would thus fill an important gap for this literature and for the wider literature on commercial product placement.

We designed and implemented a field experiment to assess the influence of a series of eight different messages, placed into three nationally-broadcast, popular Spanish-language television soap operas during the 2011–2012 broadcast season. Scenes were inserted in which characters made decisions or took action in a way that supported the message (e.g., refraining from drunk driving). After randomly selecting a five-week window during which a few scenes featuring one of the eight messages would air, we collaborated directly with the telenovela scriptwriters who wrote the scenes for each message into their respective window in one of the telenovelas. We measure unobtrusively the impact of the messages on the general U.S. Hispanic population using aggregate time series data, such as the daily tally of drunk driving arrests involving Hispanic motorists in cities with large Hispanic populations, and Google searches for message-relevant terms in Spanish and in English. We also examine self-reported behaviors using data collected by a national consumer research company, unrelated to this project. Each data series measures rates of a behavior before, during, and after the messaging, allowing us to unobtrusively test whether the messages have effects on Hispanic communities, which includes the direct effects on viewers and the secondhand effects on non-viewers. Taken together, the range of messages and data sources also provides a sense of which types of behaviors are more readily moved by embedded messages.

The objective of this study is to assess the effects of telenovela behavioral product placements at the level of the general Hispanic population in the United States. While several theories of influence and behavior change predict that placing positive behaviors into a soap opera could cause an observable ripple in behavior in the population, given that viewers like attractive and immersive stories, identify with the characters, do not feel preached to, and can subsequently influence their friends and family [6, 11, 15, 22–24], not all theories would predict a behavioral response. Other theories of media influence point out that viewers’ interpretation of media stories is personal and culturally dependent, and that many factors may cause viewers to reject the message. These factors may include viewers’ immediate social environment, their level of material resources, and the extent to which their cultural background contrasts with the media storyline [25, 26]. The results of this study can help begin to adjudicate among these contrasting predictions from different theoretical camps, and to inform pragmatic attempts to change population-level patterns using mass media.

## 1 Materials and Methods

### Setting

We collaborated with a major U.S. based Spanish-language media company with a notable history of weaving into its telenovelas messages intended to promote the well-being of Hispanic viewers. The company volunteered valuable broadcasting space to test the impact of messages aimed at improving the well-being of the Hispanic community. Its viewership, like the Hispanic population in the U.S., is growing rapidly, and the three prime-time soap operas into which our messages were embedded had an estimated viewership of 1.2 million per week on average. As a comparison, the popular Emmy-winning series *Modern Family* had an estimated weekly viewership of 5.4 million viewers during this period. Eighty-eight percent of the network’s evening viewers are foreign-born, 90% are most comfortable speaking in Spanish, and 63% have yearly household incomes of under $40,000. According to surveys of the U.S. Hispanic population, 55% of prime-time telenovela viewers are women, a much lower percentage than is typical of English-language soap opera viewers [27]. The soap operas follow the standard telenovela format: they air each weeknight for 20 weeks, feature a half-dozen primary characters, and present a mix of romantic intrigue, melodrama, and lighthearted family comedy.

### Messages

We selected messages in partnership with network executives and writers. Our priority was to choose messages that would address some of the most pressing health, financial, and social issues for Hispanics in the U.S. All proposed messages were first verified to be an issue of special concern for the U.S. Hispanic population, using recent national social demographic data. After meeting this prerequisite, the messages needed to encourage a behavior that the research team could measure across time on a national scale, and to be feasibly worked into any of three telenovela plots.

Based on these prerequisites, we selected eight messages. Three messages concerned health: eating low cholesterol foods to avoid cardiovascular disease, replacing some of the junk food served to children with healthy alternatives such as fruits and vegetables, and quitting smoking. Two safety messages concerned drunk driving and the use of child car seats. Two messages concerning viewers’ financial well-being included the availability of college scholarships for students of Hispanic heritage and the importance of saving money in banks rather than keeping cash at home. A community engagement message emphasized the importance of registering to vote. To assist the scriptwriters, we developed a factsheet underscoring the special relevance of each message for the Hispanic community (see Supplementary Information, SI). For example, the factsheet on child car seats noted that Hispanic children are twice as likely as non-Hispanic white children to die in traffic accidents. The research team did not otherwise shape the script or determine the number of scenes per message.

At face value, some messages appear to apply only to particular subpopulations within the Hispanic community—for example, men are more likely to die of heart attacks due to high cholesterol, or to be apprehended for or injured or killed in drunk driving accidents. However, the scriptwriters involved various types of characters in these behaviors so that the messages would apply to most viewers. For example, female characters were heavily involved in both the cholesterol and drunk driving messages. In the case of eating low cholesterol foods, female characters bought and cooked the lower cholesterol foods for a male character who suffered a heart attack, and in the case of drunk driving, a woman and a man both pleaded with an intoxicated friend not to take the wheel. These stories underline the point that various types of people may be involved in a behavior that is ultimately carried out among a subpopulation of the total viewership. Thus, the messaging scenes included different types of characters, and as researchers we did not limit our measurements to a hypothesized subpopulation like males or even telenovela viewers, since we were interested in determining whether the product placement effects were detectable at the level of the general Hispanic population.

All eight messages across the three telenovelas involved a total of 23 scenes in which a character or group of characters discussed or enacted the targeted behaviors, representing an accumulated 16 minutes and 51 seconds. These scenes were not central to the shows’ plots, though many involved the shows’ main characters (see SI for a detailed description of the number of scenes associated with each message and the prominence of the characters who participated in the scenes).

### Random assignment

Prior to the start of the season and before scriptwriting began, we divided each telenovela into four time periods of approximately five weeks. This gave us twelve total time periods; each of our eight chosen messages was randomly assigned to one period. This left four time periods in which the programs had no assigned message. Table 1 details each message’s assigned time period and actual air dates for each scene. Due to broadcasting constraints unrelated to the content of the messages, four scenes were aired just outside the randomly assigned time period (S1 Table). Additionally, one message (anti-smoking) was not written into the script due to a miscommunication between the research team and the scriptwriters.

**Table 1:** Messages, assigned broadcast periods, air dates, and sample outcome measurements.

Message	Telenovela	Randomly-assigned broadcast period	Air dates	Sample outcomes
Eat healthy: Lowering cholesterol	1	Episodes 36 to 60	01-23-2012; 01-30-2012; 02-06-2012	Survey Panel: % Hispanic respondents & % Hispanic prime-time telenovela viewers reporting that they watched their diet and ate fat-free, low-fat, or low cholesterol food, and that they bought low-fat food when watching their diet
				Google search term over time: “Aceite de oliva” (olive oil)
Open a bank account	1	Episodes 61 to 85	03-19-2012; 04-32-2012	Administrative data: # bank accounts opened in branches w/ Hispanic customer base, from bank featured in telenovela
				Survey Panel: “Has savings account”
				Google search term: “Banco” (bank)
Register to vote	1	Episodes 86 to 110	04-30-2012; 05-07-2012	New Hispanic voters registrated from dates January 1, 2010, to June 29, 2013.
				Survey Panel: “Reports being registered to vote”
				Google search term: “Votar” (vote), “Rock the vote”
Don’t drink and drive	2	Episodes 36 to 60	03-19-2012; 04-16-2012	Administrative: Police records of Hispanic drunk driving arrests, 7 US cities with large Hispanic populations
				Google search term: “DUI” (Driving under influence)
Seek out university scholarships	2	Episodes 61 to 85	05-21-2012; 05-28-2012; 06-04-2012	Administrative: Hits to scholarship site recommended in the plotline
				Survey Panel: “Owns 529 college savings”
				Google search term: “Beca” (scholarship)
Don’t smoke	3	Episodes 11 to 35	NA	NA
Eat healthy: Vegetables	3	Episodes 80 to 104	07-16-2012	Survey Panel: “Tries to eat healthy food or balanced diet”
				Google search term: “Verduras” (vegetables)
Use carseats for infants	3	Episodes 105 to 129	09-03-2012; 09-24-2012	Survey Panel: “Car safety features are important”
				Google search term: “Carseat”

### Outcome measures

Each message’s effects were gauged using population-level data, including at least one type of direct behavioral measurement in addition to self-reported measures, as described in Table 1. The collection of outcome measures included weekly national survey data from a consumer research firm, daily administrative data from corporations such as banks and from government sources such as police departments, and daily Internet activity data. Given our time-series experimental design, we sought outcome measures that were measured on a fine-grained individual level at a daily or weekly rate, and on a national or sub-national scale. Within these specifications, we selected a range of measures, from relatively low-effort responses to a message such as searching online for banks, to effortful responses like opening a checking account. In this way, some outcome measures provide a direct assessment of whether the message provoked the desired behavior, such as checking accounts opened, drunk driving arrests, or new voter registrations, whereas other measures gauge viewers’ interest in or information-seeking about the behavior, such as searching online for information about the behavior or stating a preference for the behavior in the context of an unrelated consumer survey. All outcomes had the advantage of unobtrusive measurement—individuals were never aware that their behavior or opinions were being tracked as part of a study of telenovela influence.

To measure online searches, we tracked search terms entered into Google through the free service Google Trends. With our partner Spanish language media company, we developed an extensive list of potential search terms in Spanish and English related to each message, and downloaded search data for the 128 terms available on Google Trends. For example, we were able to download data on the rate of Google searches for the phrase *“Aceite de oliva”* and for the word *“verduras”* (“olive oil” and “vegetables”) in Spanish and in English across the country for a two-year period spanning the time before, during and after our messages were aired. This outcome measurement strategy has been used previously by scholars seeking to gauge the impact of media programming [28]. The search terms we tracked were limited to those made available by Google Trends, which does not report search terms without a sufficiently high frequency of searching.

To measure self-reported behaviors and opinions, we relied on national survey data from a commercial consumer research firm [27]. For example, to assess the impact of the message about eating more vegetables, we amassed two years’ worth of weekly responses to the statement “I try to eat healthy food or balanced diet” from Hispanic consumers. Importantly, these data were gathered by a separate firm unconnected to our research team, without any reference to the telenovelas under study, which make the self-report data an unobtrusive measure of behaviors and opinions.

Our behavioral outcome measures were not restricted to telenovela viewers; by focusing on national outcomes among Hispanics or Spanish-speakers, we gauge whether messages conveyed to an audience of more than one million people had detectable community-wide effects. For our survey data, we test whether any effects are replicated or magnified among Hispanic respondents who also report watching prime-time telenovelas.

## 2 Results

In order to estimate the effects of the product placement treatment (*T*) on each outcome (*Y*), we use least-squares regression. For outcomes that are measured weekly, we denote each observation with an index *j* that ranges from 1 to N (the number of weeks) and regress *Y*_*j*_ on lagged outcomes *Y*_*j*−1_, an indicator variable *T*_*j*_ scored 1 if the treatment is aired in a given week, and four seasonal indicator variables:

$$\begin{array}{c} Y_{j}=\rho Y_{j-1}+\beta T_{j}+\alpha_{1}Spring_{j}+\alpha_{2}Summer_{j}+\alpha_{3}Fall_{j}+\alpha_{4}Winter_{j}+u_{j}, \end{array}$$

where *u*_*j*_ represents the unobserved disturbance term.

The key parameters of interest are *β*, the instantaneous effect of the treatment, which decays at a weekly rate of *ρ*. An alternative specification includes not only *T*_*j*_ but also *T*_*j*−1_, allowing the treatment’s behavioral effects to manifest more gradually. For this specification, we test the joint significance of *T*_*j*_ and *T*_*j*−1_.

The corresponding specification for outcomes measured on a daily basis regresses *Y*_*j*_ on outcomes lagged one day (*Y*_*j*−1_), outcomes lagged seven days (*Y*_*j*−7_), an indicator variable *T*_*j*_ scored 1 if the treatment is aired that day, and five indicator variables for each weekday. We also estimate a model that includes both immediate and lagged treatments and test their joint significance.

**Fig 1:**
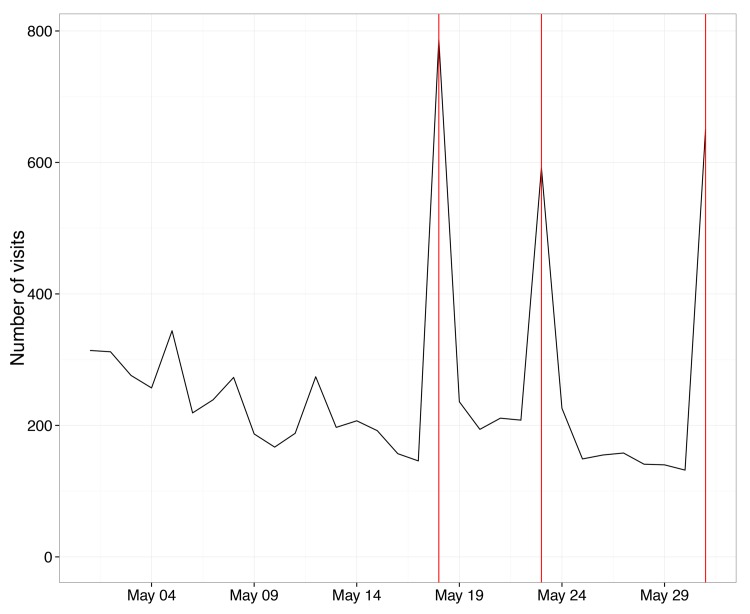
Restricted to prime-time hours, unique visits to the Hispanic Scholarship Fund, which was featured in one novela as part of the “Seek out university scholarships” message. The solid line tracks the absolute number of all visits to the website in a month-long period before, during, and after the scholarship product placement scenes, which are indicated by the vertical lines. Unique visits to the website spike on each day that the scholarship message is broadcast on the telenovela.

Our outcomes are summarized in S1, S2, S3 and S4 Figs. Two telenovela messages did significantly affect audience behavior. Fig 1 shows that following the broadcast of the “scholarship” message scenes, depicting Hispanic teens applying for scholarships (indicated by the vertical line), we see a threefold increase in visits to the scholarship website featured in the program. Time-series analysis of the daily number of Web site visits shows a highly significant treatment effect (*p* <.05), though the absolute size of this effect is small, an increase of 512 visits per day (± 149 visits). On an average non-product placement day, the website received 3,234 visits, which means that the telenovela scenes increased visits by 15%. As a comparison, consider that the Princeton University and the University of California, Los Angeles financial aid websites received an average number of 647 and 44 visits per day during the same period, respectively. The effect of the scholarship message, however, died out after one week.

**Fig 2:**
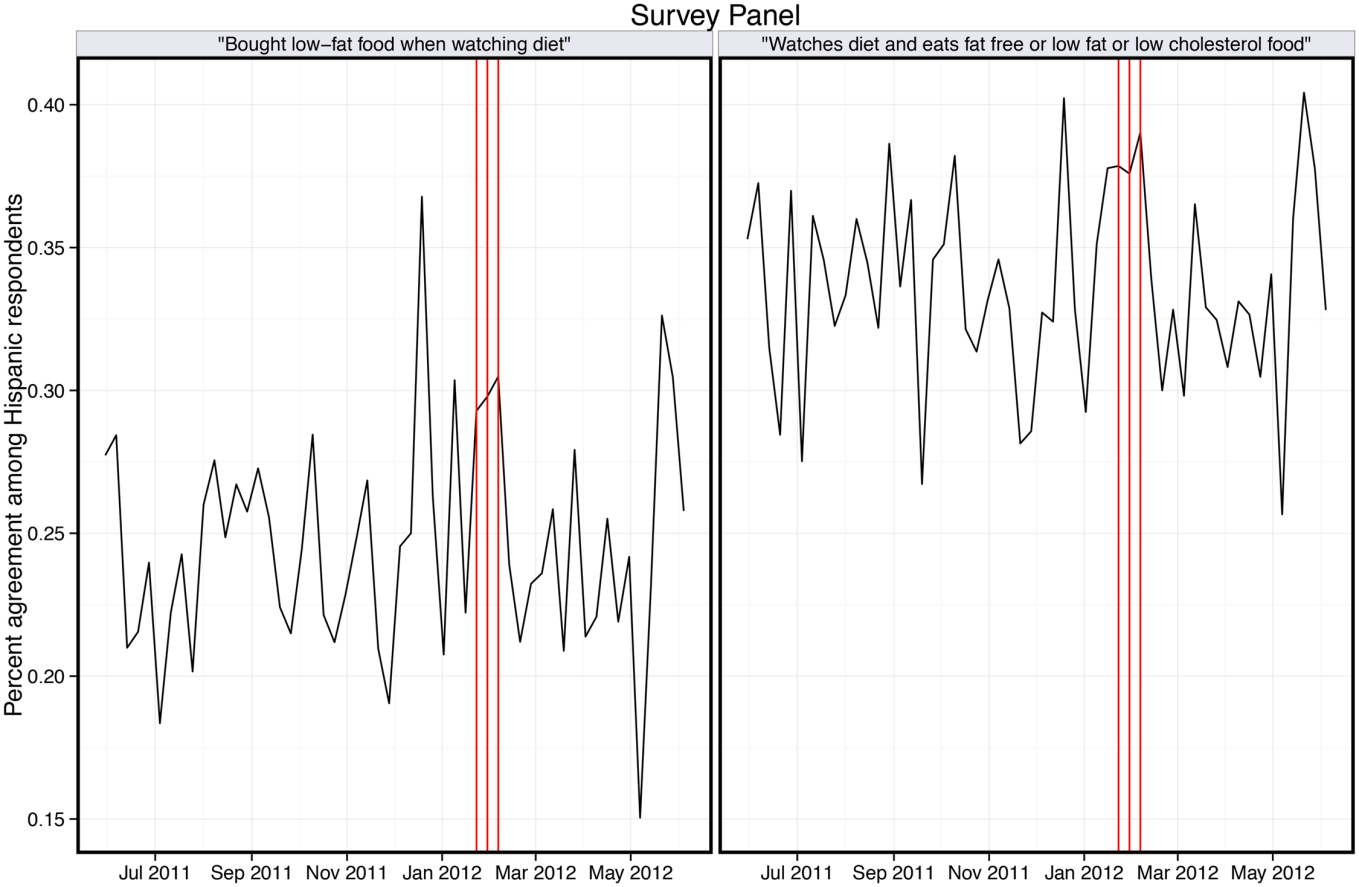
Survey data from a Hispanic respondent panel. self-reported behavioral responses related to the “Eat healthy: lower your cholesterol” message show an effect of this product placement broadcast on respondent’s propensity to watch their diet and eat fat-free, low-fat, or low cholesterol food, and buy low-fat food when watching their diet (product placement scene broadcasts are indicated by vertical lines).

**Fig 3:**
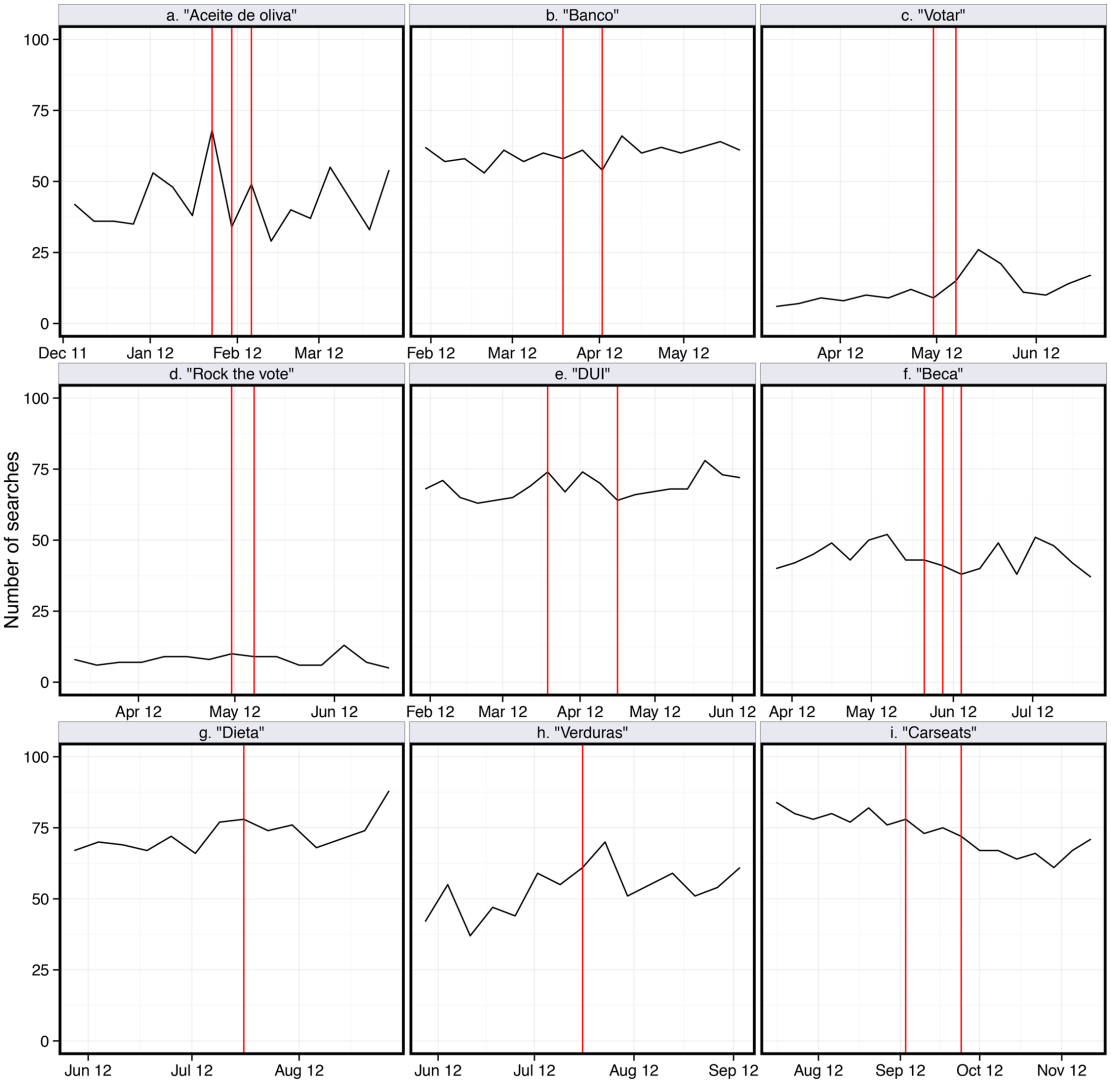
Weekly search terms on Google that relate to each experimental message. Nine figures, related to nine different messages, demonstrate that there is no detectable effect of the product placement into scenes broadcast during randomly assigned five week periods of the telenovela (product placement scene broadcasts are indicated by vertical lines). See SI for time series regression results for all 7 messages and for plots depicting these series over the course of one year.

The “Eat healthy: lower your cholesterol” messages effects were observed in terms of Google searches and in self-reported behavior. Fig 2 shows that Hispanic respondents to the survey panel became significantly more likely during the treatment period to report that they watched their diet and ate fat-free, low-fat, or low-cholesterol food, and that they bought low-fat food when watching their diet (*p* = .02 and *p* = .009). When testing for the joint significance of the treatment and the one-week lagged treatment, we find a similar pattern (*p* = .05; see S2 and S3 Tables). Additionally, Fig 3a shows that Google searches for the term “aciete de oliva” (olive oil, a substitute fat suggested by the telenovela’s doctor character as part of that message) rose during the airing of the cholesterol message (*p* = .02, when testing for the joint significance of the immediate and one-week lagged treatment, S3 Table). All findings remained significant when testing only the subpopulation of Hispanic individuals who identified as prime-time telenovela watchers. That these specific survey responses moved during the air dates of the message and not related responses that were not addressed by the telenovela plot (such as “going on a diet”) gives us some confidence in these results, even though the effects are small and short-lived. Moreover, these positive results were anticipated a priori, given that the “eat lower cholesterol foods” message was one of the most well-integrated messages of all eight product placements, in terms of the number of appearances of the messages and the involvement of primary characters in the messaging. Nevertheless, these positive results must be discounted to some extent, given the sheer number of tests (seven Google Trends terms, and sixteen survey items) that do not show significant effects in this domain.

**Fig 4:**
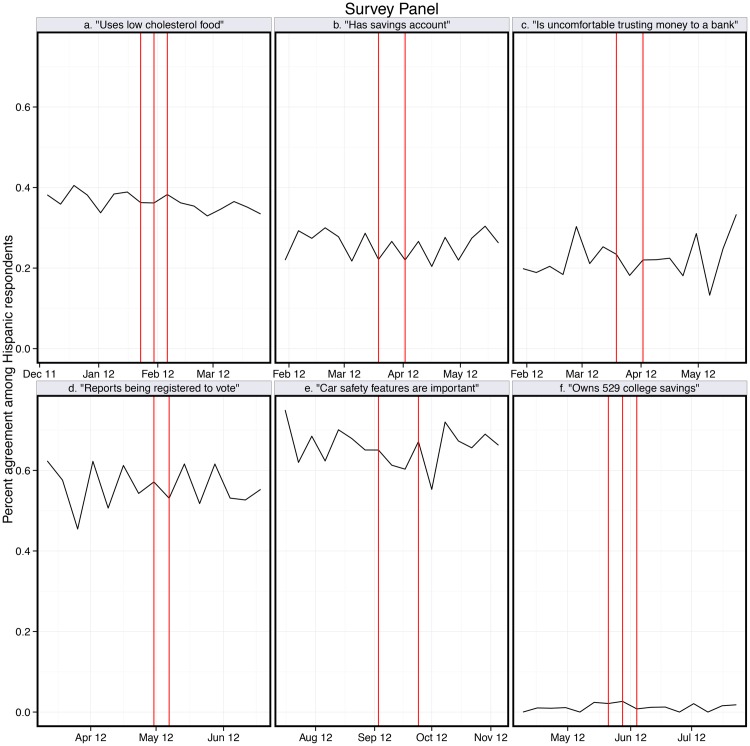
Survey data from a Hispanic respondent panel. attitudinal and self-reported behavioral responses related to each experimental message. Six figures, related to six different messages, demonstrate that there is no detectable effect of the product placement into scenes broadcast during randomly assigned five week periods of the telenovela (the broadcast of the product placement is indicated by vertical lines). See SI for time-series regression results for all 7 messages and for plots depicting one year of data (as opposed to 3 months, presented here for ease of visual inspection).

The behavioral effects were observed in instances when viewers could enact a relatively lower-effort behavior immediately following or during viewing, in this case searching online for information or for a scholarship resource. We do not find any other significant impacts of the messaging on other low-effort behavioral responses, mainly Google searching, as summarized in Fig 3. The panel survey data from Hispanic consumers, depicted in Fig 4 and in S2 and S3 Tables, show no other changes in self-reported behaviors, such as owning an ATM or debit card. These results are based on responses from adults who identify as Hispanic or Latino/a, and our results do not change when we further narrow the population pool to those who report viewing telenovelas.

**Fig 5:**
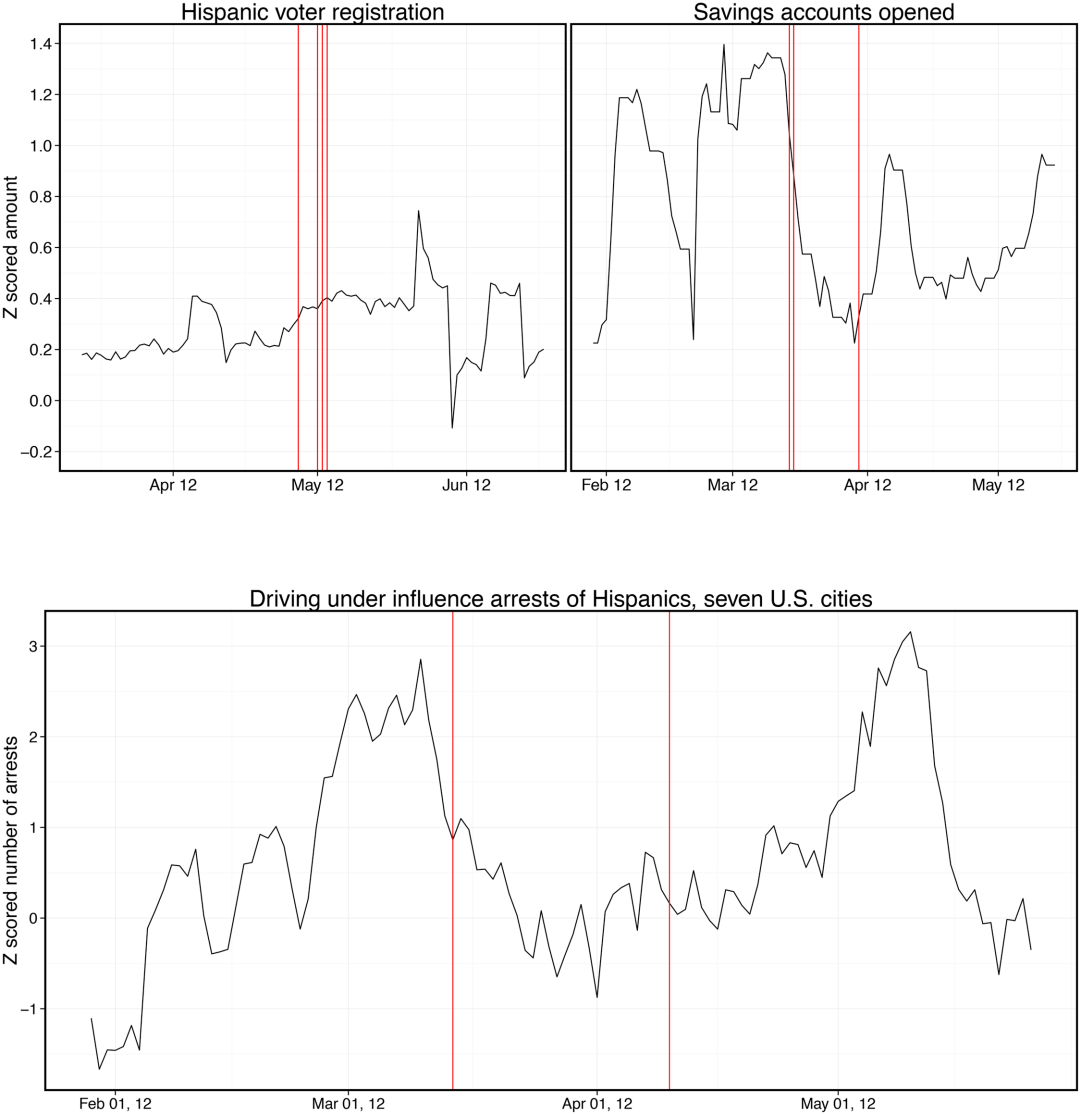
Administrative data charting three behaviors over the course of three months before and after randomly-assigned telenovela product placement. The top figure depicts numbers of arrests of Hispanic individuals for driving under the influence, z-scored and averaged across 7 major U.S. cities. The occurrences of product placement scenes depicting a “Don’t drink and drive” message are represented by the figure’s vertical lines. The bottom two figures show number of Hispanics who registered to vote over time, and the number of savings accounts opened at Hispanic consumer base branches of the bank that was featured in the telenovela. Those two figures test the efficacy of the “Register to vote” and “Open a bank account” messages, inserted into the randomly assigned broadcast periods as indicated by vertical lines. All three figures show negligible effects of the product placement on these behaviors. See SI for time-series regression results and for plots depicting one year of data as opposed to 3 months (presented here for ease of visual inspection).

Behaviors requiring more effort beyond the immediate viewing session, such as opening a bank account or registering to vote, are unresponsive to embedded messaging. Fig 5 charts the total number of new accounts opened with a regional bank whose name was featured during one of the banking product placement scenes. Restricting attention to the 328 branches with Spanish-speaking tellers, the time series does not respond immediately or in a delayed fashion to the banking scenes. We observed the same null effects for other outcomes that also require the viewer to take actions or change behaviors after the episode had ended. For example, Fig 5 also depicts how telenovela messaging has no apparent effect on the daily number of voter registrations by Hispanics, nor does it reduce the number of drunk driving arrests of Hispanic motorists in seven major urban areas.

Our randomization facilitates a test for intent-to-treat effects that requires minimal statistical assumptions. We predict a standardized weekly behavioral outcome (such as rates of Google searching for each of the seven messages) using the seven randomization periods, rather than the precise dates that the scenes were broadcast, which was not directly randomly assigned. Here too we find no evidence of an overall treatment effect (see the SI for this and other more extensive analyses).

Lest these null findings provoke speculation that our design lacks power to detect product placement effects, we would point out that our topic-level regressions often have quite small standard errors. For example, to achieve 80% power, the expected t-ratio must exceed 2.5, which in the case of drunk driving amounts to just 11.5 fewer Hispanic daily arrests across the seven cities for which we have data. Similarly, our test would have had 80% power if the 328 Spanish-speaking branches of the bank featured in the telenovelas had jointly experienced an increase of 400 new bank accounts during the week following the dramatization.

## 3 Discussion

Our statistical results indicate, perhaps surprisingly, that while two of the behavioral product placement messages have a statistically significant effect, they were short-lived. Beyond the fragility of these findings, the behaviors that were affected did not require the exertion of effort beyond the most immediate viewing context. Finally, no message generated effects that are large or sustained enough to be policy-relevant. We conclude by discussing the theoretical implications of these results, which stand in contrast to the strong positive findings that often emerge from evaluations of mass media messaging.

It is theoretically informative to contrast our findings with those from several recent field experimental studies of the influence of education-entertainment soap operas, which are thematically focused on particular behavioral messages. These studies have found strong effects in the wake of entertainment series in which the main plot is oriented around a social message [17–19]. These effects are predicted by theories of entertainment-education [15], social and narrative learning [6, 7, 23], parasocial interaction [10], and by the elaboration-likelihood model of persuasion [9], which describe the conditions for behavior change as those in which media is attractive and immersive, viewers can relate to or aspire to be the characters, and messages are embedded so naturally in the plot line that viewers do not necessarily recognize them as direct attempts at persuasion. However, several media theories elaborate the circumstances under which these aspects of media influence may not be sufficient. Audience reception theory and related ethnographic accounts of media influence [25, 26, 29] emphasize that media viewing is interpretive and that the effects of viewing can be personally idiosyncratic and culturally dependent. Depending on viewers’ level of education, social position, and other forms of “cultural capital,” they may reject, embrace, identify, or resist messages [26, 29]. With particular relevance to our sample, studies have revealed that viewers from lower-income backgrounds may be used to steeling themselves against messages that involve economic costs [25]. Many of the messages in the present study did encourage behaviors that were somewhat costly, including the cost of time if not direct financial costs (for example, taking time to register to vote). Additionally, other studies guided by this theoretical perspective have shown that viewers who are not native to the media environment may resist acculturation messages by maintaining ties with media from home [30], which may be relevant to the telenovela viewers, of which 88% were born outside of the U.S.

Other media scholars who predict small to null population-level media effects note the “noise” of the present-day media environment, meaning both the vast array of available media programming available to viewers and the numerous distractions that accompany a typical media viewing setting [11]. In our study, it is notable that the messages and resulting product placement scenes make up a small percentage of the total air time and are incidental to the broader themes of the telenovelas. As a result, the messages are repeated less often than would be the case in a dedicated education-entertainment program that has been found to produce significant behavioral effects in other field experiments. Although intermittent messages like ours may be less likely to provoke audience resistance, a positive feature under the elaboration likelihood model [9], they also may be less likely to be noticed, and less likely to inspire the audience to make a lifestyle-altering decision such as opening a bank account or changing diet.

An encompassing theory of media influence must grapple with the emerging distinction between modeled incidental behavior and modeled behavior that follows from the series’ broader theme. While one study cannot adjudicate among various theoretical perspectives on media influence, our results suggest at least some boundary conditions on the positive predictions of the entertainment-education, social modeling, and elaboration-likelihood theories of influence and related theories. Even when the story is immersive or characters are beloved, the behaviors may need to be featured more regularly and may need to be more central to the theme of the story in order to observe significant behavioral influence. Occasional behavioral product placement may be too brief to be noticed, or it may seem too extraneous to be influential. We cannot determine whether the behavioral product placements in this study were resisted or outright rejected, according to the predictions of media acculturation theory, but we can say that theories predicting positive media influence may not extend to the case of behavioral product placement that is relatively brief, as it was in our case.

Our findings also underline the importance of solid empirical data to inform this theoretical debate. The time series experimental design used here can be used to study causality in national mass media broadcasting, which has heretofore been studied with correlational designs that provide much weaker evidence regarding the effects of various types of product placement. In particular, our design can be used to evaluate the strong claims by corporations and media companies that brief product placements, even for one episode, can boost sales (as exemplified by citations of increased Pottery Barn sales following its appearance in one episode of the television series *Friends* [2]. As Balasubramanian et al. [5] note in their extensive literature review, few studies to date have combined experimental designs and unobtrusive behavioral outcome measures to assess the cost-effectiveness of product placement. Previous experimental studies of product placement have typically presented media messages in lab settings and have focused on intermediate outcomes, such as brand awareness or evaluation.

In our study, the airtime devoted to the suite of messages would have been worth millions of dollars, but the cumulative effect of these messages on the general population was small and short-lived. Other product placement campaigns have focused on making the desired behavior, such as the purchase of Reese’s Pieces, extremely accessible by placing the candies in giant displays at the movie theaters where *E.T*., the film that famously featured the candy, was playing. Such “integrated marketing communications” [5] may make it easier for product placement campaigns to have an effect, as predicted by psychological theorizing about the pragmatic behavioral channels that facilitate action from motivation [31]. Further research using the present style of field experimental design is needed to establish whether other kinds of product placement strategies could have an effect on a population’s behavior.

## Supporting Information

S1 Table Messages, randomly-assigned broadcast period, and sample outcome measurement. The eight messages randomly assigned to be broadcast within a five-week time period, in one of three possible telenovelas. Each telenovela was divided into four time periods of approximately five weeks, excluding four weeks of introduction to the telenovela, during which characters and plots were developed. The table indicates to which telenovela of three each message was assigned, and the dates of that time period (the telenovelas were broadcast in sequence from first to third, but overlapped in time). All outcome data gathered to measure each variable are listed in the final column; most data were gathered over a timespan of three years, depending on availability. Three messages were broadcast late, after the end of the randomization window, for arbitrary reasons related to the telenovela’s broadcasting schedule. They were: one scene featuring the “Open a bank account” message, aired 3 days after the assigned time-frame had ended, two scenes for the the “Seek out university scholarships” message, aired 1 and 7 days after the assigned time-frame had ended, and one of the scenes for the “Use carseats for infants” message, aired 11 days after the assigned time-frame had ended.
(PDF)

S2 Table Estimated effect of the telenovela product placement on each outcome measure, and the descriptive characteristics of each outcome for the total sample across time. a = Estimated average effect of the randomly-timed telenovela product placement scenes on the measured outcome, controlling for the one-day or -week (depending on the frequency of the outcome measure) lag of the outcome, and for the fixed effects of day of the week or for week of the month (depending on the frequency of the outcome measure). b = Standard error of the estimated average effect of the telenovela scene on the measured outcome. c = Mean of the outcome for the total sample across time. d = Range of all possible values (min-max) for outcome. e = Standard deviation of the outcome for the total sample across time. f = Estimated average effect of the randomly-timed telenovela product placement scenes on the one-day lag of the measured outcome, for the fixed effects of day of the week or for week of the month (depending on the frequency of the outcome measure). g = Standard error of the estimated average effect of the telenovela scene on the one-day lag of the measured outcome.. = *p* < .1; * = *p* < .05; ** = *p* < .01; *** = *p* < .001.
(PDF)

S3 Table Estimated effect of the telenovela product placement on each outcome measure, and the descriptive characteristics of each outcome for the total sample across time. a = Estimated average effect of the randomly-timed telenovela product placement scenes on the measured outcome, controlling for the one-day or -week (depending on the frequency of the outcome measure) lag of the outcome, for the lag of the treatment period (the previous broadcast), and for the fixed effects of day of the week or for week of the month (depending on the frequency of the outcome measure). b = Standard error of the estimated average effect of the telenovela scene on the measured outcome. c = Estimated average treatment effect of the one-period lag of the telenovela product placement on the measured outcome. d = Standard error of the estimated average treatment effect of the lagged telenovela product placement on the measured outcome. e = Mean of the outcome for the total sample across time. f = Range of the outcome in the total sample across time (min-max). g = Standard deviation of the outcome for the total sample across time. h = *p*-value of an F-test of the joint significance of the immediate and lagged effect of the telenovela treatment on the outcome.
(PDF)

S4 Table Randomization-based intention to treat analysis: googling about any of the seven messages, predicted by the five-week period to which the message was randomized. This analysis uses the five-week period, the directly-randomized treatment unit, as the predictor of whether Googling about the topics of the broadcast message increased during that time period. The dependent measure is a standardized Google trends score per week, for “aceite de oliva,” “banco,” “voto,” “DUI,” “beca,” “verduras,” and “carseat.” We include fixed effects controls for each period, a control variable for week, calculate standard errors clustered by treatment unit.
(PDF)

S1 Fig Restricted to prime-time hours, unique visits to the Hispanic Scholarship Fund, which was featured in one novela as part of the “Seek out university scholarships” message. The solid line tracks the absolute number of all visits to the website in a month-long period before, during, and after the scholarship product placement scenes, which are indicated by the vertical lines. Unique visits to the website spike on each day that the scholarship message is broadcast on the telenovela. No such spikes in visitor numbers are observed on these days in the previous year, indicated by the dashed line.
(TIF)

S2 Fig One year of weekly search terms on Google that relate to each experimental message. Nine figures, related to nine different messages, demonstrate that there is no detectable effect of the product placement into scenes broadcast during randomly assigned five week periods of the telenovela (the broadcast of the product placement is indicated by vertical lines).
(TIF)

S3 Fig One year of survey data from a Hispanic respondent panel: attitudinal and self-reported behavioral responses related to each experimental message. Six figures, related to six different messages, demonstrate that there is no detectable effect of the product placement into scenes broadcast during randomly assigned five week periods of the telenovela (the broadcast of the product placement is indicated by vertical lines).
(TIF)

S4 Fig One year of administrative data charting three behaviors over time, before and after randomly-assigned telenovela product placement. The top figure depicts numbers of arrests of Hispanic individuals for driving under the influence, z-scored and averaged across 7 major U.S. cities, over time. The occurrence of scenes depicting a “Don’t drink and drive” message is represented by the figure’s vertical lines. The bottom two figures show number of Hispanics who registered to vote over time, and the number of savings accounts opened at Hispanic consumer base branches of the bank that was featured in the telenovela. Those two figures test the efficacy of the “Register to vote” and “Open a bank account” messages, inserted into the randomly assigned broadcast periods as indicated by vertical lines. All three figures show negligible effects of the product placement on these behaviors.
(TIF)

S1 Text Description of messages. (PDF)

S2 Text Factsheets given to scriptwriters. (PDF)

S1 Link Replication code and data. Web version of the figures.
(DOCX)
