# Supplementary material for: Does Product Placement Change Television Viewers’ Social Behavior?
Source: PLoS One. 2015 Sep 23;10(9):e0138610. doi: 10.1371/journal.pone.0138610 (PMC4580471; doi:10.1371/journal.pone.0138610)
Supplement: S1 Table — The eight messages randomly assigned to be broadcast within a five-week time period, in one of three possible telenovelas. Each telenovela was divided into four time periods of approximately five weeks, excluding four weeks of introduction to the telenovela, during which characters and plots were developed. The table indicates to which telenovela of three each message was assigned, and the dates of that time period (the telenovelas were broadcast in sequence from first to third, but overlapped in time). All outcome data gathered to measure each variable are listed in the final column; most data were gathered over a timespan of three years, depending on availability. Three messages were broadcast late, after the end of the randomization window, for arbitrary reasons related to the telenovela’s broadcasting schedule. They were: one scene featuring the “Open a bank account” message, aired 3 days after the assigned time-frame had ended, two scenes for the the “Seek out university scholarships” message, aired 1 and 7 days after the assigned time-frame had ended, and one of the scenes for the “Use carseats for infants” message, aired 11 days after the assigned time-frame had ended. (PDF) [file pone.0138610.s001.pdf]

Message	Telenovela	Randomly-assigned broadcast	Type	Variable measured
Eat healthy: Lowering cholesterol	1	19-01-2012 to 03-02-2012	Survey Panel	“Presently watching diet”
				“Watches diet and eats fat free or low fat or low cholesterol food”
				“Reason for watching diet is cholesterol”
				“Uses butter”
				“Uses eggs ”
				“Bought low cholesterol food when watching diet”
				“Uses low-fat/fat-free products”
				“Bought low-fat food when watching diet”
				“Eats margarine”
				“Eats mayonnaise”
				“Uses salad/cooking oil”
				“Uses olive oil”
				“Does not have cholesterol but feels at risk”
				“Reports using shortenings”
			Google Searching behavior	“Bajar el colesterol”
				“Dieta”
				“Aceite de oliva”
				“Clara de huevo”
				“Colesterol”
				“Egg yolks”
				“Egg whites”
Open a bank account	1	14-03-2012 to 30-03-2012	Administrative	Number of bank accounts opened
			Survey Panel	“Has ATM or Debit card”
				“Often pays things in cash”
				“Has a credit card”
				“Is uncomfortable trusting money to a bank”
				“Has vehicle or property insurance”
				“Is not good at saving money”

				"Has savings account"
			Google Searching behavior	"Banco"<br>"Como abrir una cuenta"<br>"Robo"<br>"BB&T"<br>"Bank"
Register to vote	1	27-04-2012 to 03-05-2012	Administrative	Voting registration by Hispanics, 01-01-2010 to 06-29-2013
			Survey Panel	"It is important to be informed to vote"<br>"Reports being a member of any club or organization listed"<br>"Reports being registered to vote"
			Google Searching behavior	"Rock the vote"<br>"Eleccion"<br>"Voto"<br>"Votar"
Don't drink and drive	2	14-03-2012 to 10-04-2012	Administrative	Drunk driving arrests of Hispanics in 7 US cities
			Google Searching behavior	"DUI"<br>"Borracho"
Seek out university scholarships	2	18-05-2012 to 31-05-2012	Administrative	Hits to Scholarship site recommended in the plotline
			Survey Panel	"Owns 529 college savings "<br>"Owns other college savings "
			Google Searching behavior	"Yale"<br>"Beca"<br>"Universidad"<br>"Prestamo"<br>"Diseño gráfico"
Eat healthy: vegetables	3	12-07-2012 to 12-07-2012	Survey Panel	"Works on eating a well balanced diet"<br>"Bought low-calorie foods when watching diet"

				“Eats food regardless of calories”<br>“Presently watching diet”<br>“Tries to eat healthy food or balanced diet”<br>“Tries to include more fiber into diet”<br>“Looks for freshest ingredients when cooking”<br>“Uses fresh produce”<br>“Tries to eat healthier foods these days”<br>“Uses bagged/ packaged salad”<br>“Eats frozen vegetables”
			Google Searching behavior	“Papas fritas”<br>“Sobrepeso”<br>“Uvas”<br>“Mangos”<br>“Leche”<br>“Manzanas”<br>“Verduras”
Don’t smoke	3	***Not implemented*	NA	NA
Use carseats for infants	3	28-08-2012 to 21-09-2012	Survey Panel	“Car safety features are important”<br>“Looked up car safety rating before purchase”<br>“Regularly wears seatbelt”
			Google Searching behavior	“Accidente automovilistico”<br>“Carseat”
