# Supplementary material for: Does Product Placement Change Television Viewers’ Social Behavior?
Source: PLoS One. 2015 Sep 23;10(9):e0138610. doi: 10.1371/journal.pone.0138610 (PMC4580471; doi:10.1371/journal.pone.0138610)
Supplement: S2 Table — a = Estimated average effect of the randomly-timed telenovela product placement scenes on the measured outcome, controlling for the one-day or -week (depending on the frequency of the outcome measure) lag of the outcome, and for the fixed effects of day of the week or for week of the month (depending on the frequency of the outcome measure). b = Standard error of the estimated average effect of the telenovela scene on the measured outcome. c = Mean of the outcome for the total sample across time. d = Range of all possible values (min-max) for outcome. e = Standard deviation of the outcome for the total sample across time. f = Estimated average effect of the randomly-timed telenovela product placement scenes on the one-day lag of the measured outcome, for the fixed effects of day of the week or for week of the month (depending on the frequency of the outcome measure). g = Standard error of the estimated average effect of the telenovela scene on the one-day lag of the measured outcome.. = p < .1; * = p < .05; ** = p < .01; *** = p < .001. (PDF) [file pone.0138610.s002.pdf]

Message	Type	Variable measured	Estimate[a]	SE[b]	Mean[c]	Range[d]	SD[e]	Estimate Lag. DV[f]	SE Lag. DV[g]
Eat healthy: Lowering cholesterol	Survey Panel: weekly	“Presently watching diet”	0.035	0.027	0.46	0.34-0.58	0.04	-0.02	0.06
		“Watches diet and eats fat free or low fat or low cholesterol food”	0.06*	0.025	0.329	0.18-0.44	0.042	-0.10	0.07
		“Reason for watching diet is cholesterol”	0.007	0.019	0.158	0.07-0.25	0.033	-0.046	0.06
		“Uses butter”	0.015	0.027	0.855	0.73-0.93	0.044	0.064	0.094
		“Uses eggs”	0.011	0.021	0.943	0.83-1	0.035	0.12	0.09
		“Bought low cholesterol food when watching diet”	-0.002	0.019	0.146	0.06-0.26	0.033	-0.17	0.063
		“Uses low-fat/fat-free products”	0.072	0.047	0.51	0.34-0.76	0.103	0.63	0.08
		“Bought low-fat food when watching diet”	0.062**	0.023	0.238	0.07-0.37	0.04	-0.011	0.063
		“Eats margarine”	-0.050	0.034	0.729	0.6-0.88	0.056	0.18	0.09
		“Eats mayonnaise”	-0.037	0.024	0.846	0.72-0.93	0.039	0.044	0.095
		“Uses salad/cooking oil”	-0.0001	0.021	0.909	0.82-0.99	0.035	0.12	0.095
		“Uses olive oil”	-0.016	0.019	0.087	0.02-0.18	0.032	0.12	0.094
		“Does not have cholesterol but feels at risk”	-0.015 .	0.008	0.025	0-0.07	0.015	0.08	0.06
		“Reports using shortenings”	-0.0001	0.021	0.909	0.82-0.99	0.035	0.12	0.09
	Google searching behavior: weekly	”Bajar el colesterol”	1.95	8.38	61.98	31-100	15.127	0.33	0.10
		“Dieta”	-4.20	4.68	75.559	39-100	12.941	0.78	0.07
		“Aceite de oliva”	-1.22	8.459	62.588	29-100	17.5	0.52	0.09
		“Clara de huevo”	1.44	10.47	39.931	0-100	25.725	0.74	0.07
		“Colesterol”	-1.35	4.70	73.029	45-100	10.826	0.61	0.08
		“Egg yolks”	-3.85	5.18	26.461	17-100	9.772	0.47	0.09
		“Egg whites”	1.76	4.14	74.843	54-100	9.527	0.54	0.08
Open a bank account	Administrative: weekly	Number of bank accounts opened	-106.20	157.76	1863	994-2681	433.84	0.86	0.05
	Survey Panel: weekly	“Has ATM / Debit card”	0.026	0.042	0.596	0.44-0.74	0.063	0.32	0.06
		“Often pays things in cash”	-0.029	0.035	0.528	0.4-0.67	0.049	0.11	0.06
		“Has a credit card”	0.005	0.037	0.488	0.32-0.61	0.052	0.14	0.06
		“Is uncomfortable trusting money to a bank”	-0.002	0.03	0.23	0.13-0.38	0.041	-0.05	0.06
		“Has vehicle or property insurance”	0.034	0.037	0.645	0.51-0.78	0.052	0.14	0.06
		“Is not good at saving money”	-0.005	0.032	0.327	0.2-0.44	0.045	0.11	0.06
		“Has savings account”	-0.05	0.034	0.272	0.16-0.41	0.048	0.065	0.06
	Google searching behavior: weekly	“Banco”	-6.103	3.698	71.755	53-100	10.75	0.80	0.06
		“Como abrir una cuenta”	2.006	8.806	56.696	32-91	12.936	0.24	0.10
		“Robo”	-0.734	4.535	77.02	63-100	6.716	0.42	0.10
		“BB&T”	-0.241	3.211	81.843	72-100	4.769	0.46	0.09
		“Bank”	-0.705	2.393	92.304	79-100	3.773	0.48	0.09

Register to vote	Administrative: daily (log)	Voter registration by Hispanics, 01-01-2010 to 06-29-2013	0.065	0.30	7.378	0-11.64	1.504	0.42	0.02
	Administrative: weekly (log)	Voter registration by Hispanics, 01-01-2010 to 06-29-2013	0.100	0.335	9.626	6.27-12.13	1.032	0.98	0.03
	Survey Panel: weekly	"It is important to be informed to vote"	0.0001	0.031	0.772	0.66-0.88	0.042	0.05	0.07
		"Reports being a member of any club / organization listed"	-0.008	0.034	0.327	0.2-0.44	0.047	-0.04	0.07
		"Reports being registered to vote"	0.010	0.042	0.536	0.35-0.67	0.059	0.12	0.06
	Google searching behavior: weekly	"Rock the vote"	2.104	8.286	9.569	0-100	15.372	0.60	0.08
		"Eleccion"	-2.979	10.03	10.167	3-100	13.571	0.09	0.10
		"Voto"	-2.075	9.49	14.578	5-100	14.532	0.53	0.11
		"Votar"	-2.06	9.33	16.608	4-100	14.358	0.44	0.09
Don't drink and drive	Administrative: daily	Drunk driving arrests of Hispanics in 7 US cities	-0.829	4.639	27.315	1-72	13.933	0.19	0.02
	Administrative: weekly	Drunk driving arrests of Hispanics in 7 US cities	-13.782	14.84	191.12	119-272	24.31	0.44	0.07
	Google searching behavior: weekly	"DUI"	0.446	4.569	68.578	56-96	6.83	0.40	0.09
		"Borracho"	-2.355	5.026	67.951	45-100	12.83	0.81	0.07
Seek out university scholarships	Administrative: daily	Hits to Scholarship site recommended in the plotline	403.521	704.12	3232.8	0-22958	4315.584	0.91	0.02
	Survey Panel: weekly	"Owns 529 college savings"	0.007	0.006	0.011	0-0.04	0.009	0.06	0.06
		"Owns other college savings"	0.006	0.005	0.009	0-0.03	0.008	0.04	0.07
	Google searching behavior: weekly	"Yale"	2.625	3.074	60.608	49-100	7.548	0.57	0.08
		"Beca"	-3.47	4.813	51.873	24-91	14.95	0.84	0.05
		"Universidad"	-3.089	3.744	79.137	56-100	8.341	0.49	0.09
		"Prestamo"	-9.026	6.96	55.216	24-100	14.304	0.50	0.09
		"Diseño gráfico"	-13.03	7.95	53.941	28-98	13.802	0.11	0.10
Eat healthy:	Survey Panel: weekly	"Agrees that works on eating a well balanced diet"	-0.027	0.049	0.555	0.41-0.69	0.049	-0.08	0.06
Vegetables		"Bought low-calorie foods when watching diet"	0.087**	0.032	0.153	0.08-0.25	0.032	-0.11	0.06
		"Agrees that eats food regardless of calories"	0.041	0.047	0.498	0.36-0.61	0.046	0.02	0.06
		"Presently watching diet"	0.008	0.045	0.457	0.34-0.58	0.045	-0.01	0.06
		"Agrees that tries to eat healthy food / balanced diet"	-0.027	0.049	0.555	0.41-0.69	0.049	-0.07	0.063

		“Agrees that tries to include more fiber into diet”	0.003	0.05	0.644	0.48-0.76	0.049	0.06	0.06
		“Agrees that looks for freshest ingredients when cooking”	0.03	0.046	0.594	0.48-0.73	0.045	0.05	0.06
		“Uses fresh produce”	0.006	0.032	0.919	0.81-0.98	0.031	0.10	0.09
		“Agrees that tries to eat healthier foods these days”	0.071	0.054	0.605	0.42-0.76	0.054	0.05	0.06
		“Uses bagged/ packaged salad”	0.022	0.061	0.659	0.46-0.8	0.059	0.02	0.10
		“Eats frozen vegetables”	0.028	0.064	0.662	0.5-0.82	0.064	0.22	0.09
	Google searching behavior: weekly	“Papas fritas”	2.20	15.95	61.912	34-100	16.161	0.34	0.10
		“Sobrepeso”	-10.17	16.727	48.559	0-100	22.142	0.66	0.08
		“Uvas”	2.892	7.236	36.725	19-58	8.603	0.50	0.09
		“Mangos”	8.934	8.391	71.176	36-100	16.165	0.74	0.08
		“Leche”	2.487	7.101	80.118	53-100	7.399	0.41	0.10
		“Manzanas”	-5.187	10.164	51.373	27-100	14.397	0.61	0.08
		“Verduras”	4.571	10.977	58.059	0-97	15.339	0.77	0.08
Don’t smoke	***Not implemented*	NA	NA	NA	NA	NA	NA	NA	NA
Use carseats for infants	Survey Panel: weekly	“Car safety features are important”	-0.002	0.023	0.67	0.55-0.75	0.031	0.11	0.06
		“Looked up car safety rating before purchase”	-0.005	0.036	0.45	0.3-0.58	0.05	0.03	0.06
		“Regularly wears seatbelt”	-0.015	0.037	0.69	0.51-0.81	0.05	0.17	0.06
	Google searching behavior: weekly	“Accidente automovilistico”	-6.626	13.35	30.265	0-100	30.64	0.82	0.06
		“Carseat”	1.406	3.108	74.392	58-100	7.012	0.62	0.08
