# Supplementary material for: Does Product Placement Change Television Viewers’ Social Behavior?
Source: PLoS One. 2015 Sep 23;10(9):e0138610. doi: 10.1371/journal.pone.0138610 (PMC4580471; doi:10.1371/journal.pone.0138610)
Supplement: S3 Table — a = Estimated average effect of the randomly-timed telenovela product placement scenes on the measured outcome, controlling for the one-day or -week (depending on the frequency of the outcome measure) lag of the outcome, for the lag of the treatment period (the previous broadcast), and for the fixed effects of day of the week or for week of the month (depending on the frequency of the outcome measure). b = Standard error of the estimated average effect of the telenovela scene on the measured outcome. c = Estimated average treatment effect of the one-period lag of the telenovela product placement on the measured outcome. d = Standard error of the estimated average treatment effect of the lagged telenovela product placement on the measured outcome. e = Mean of the outcome for the total sample across time. f = Range of the outcome in the total sample across time (min-max). g = Standard deviation of the outcome for the total sample across time. h = p-value of an F-test of the joint significance of the immediate and lagged effect of the telenovela treatment on the outcome. (PDF) [file pone.0138610.s003.pdf]

Message	Type	Variable measured	Estimate[a]	SE[b]	Lag. Est.[c]	Lag. SE[d]	Mean[e]	Range[f]	SD[g]	p-value[h]
Eat healthy: Lowering cholesterol	Survey Panel: weekly	“Presently watching diet”	0.007	0.034	0.04	0.03	0.45	0.34-0.58	0.045	0.21
		“Watches diet and eats fat free or low fat or low cholesterol food”	0.05	0.033	0.012	0.033	0.329	0.18-0.44	0.042	0.075
		“Reason for watching diet is cholesterol”	0.012	0.026	-0.007	0.026	0.158	0.07-0.25	0.033	0.896
		“Uses butter”	0.013	0.035	0.003	0.035	0.855	0.73-0.93	0.044	0.857
		“Uses eggs”	0.014	0.026	-0.003	0.026	0.943	0.83-1	0.035	0.842
		“Bought low cholesterol food when watching diet”	-0.004	0.025	0.002	0.025	0.146	0.06-0.26	0.033	0.988
		“Uses low-fat/fat-free products”	0.007	0.058	0.11	0.057	0.51	0.34-0.76	0.103	0.052
		“Bought low-fat food when watching diet”	0.059	0.031	0.006	0.032	0.238	0.07-0.37	0.04	0.034
		“Eats margarine”	-0.073	0.044	0.036	0.044	0.729	0.6-0.88	0.056	0.246
		“Eats mayonnaise”	-0.056	0.031	0.031	0.031	0.846	0.72-0.93	0.039	0.20
		“Uses salad/cooking oil”	-0.018	0.027	0.029	0.027	0.909	0.82-0.99	0.035	0.551
		“Uses olive oil”	-0.037	0.025	0.033	0.025	0.087	0.02-0.18	0.032	0.303
		“Does not have cholesterol but feels at risk”	-0.017	0.012	0.003	0.012	0.025	0-0.07	0.015	0.205
		“Reports using shortenings”	-0.018	0.027	0.029	0.027	0.909	0.82-0.99	0.035	0.551
	Google searching behavior: weekly	”Bajar el colesterol”	6.523	11.141	-6.82	10.92	61.98	31-100	15.13	0.801
		“Dieta”	1.065	6.22	-7.93	6.21	75.559	39-100	12.941	0.299
		“Aceite de oliva”	19.013	10.80	-30.80	10.77	62.59	29-100	17.5	0.020
		“Clara de huevo”	3.654	13.88	-3.37	13.79	39.931	0-100	25.73	0.812
		“Colesterol”	1.047	6.28	-3.62	6.26	73.029	45-100	10.826	0.813
		“Egg yolks”	-4.608	6.91	1.16	6.92	26.461	17-100	9.772	0.751
		“Egg whites”	4.788	5.50	-4.60	5.49	74.843	54-100	9.527	0.644
Open a bank account	Administrative: weekly	Number of bank accounts opened	-104.751	158.4	52.15	157.58	1863	994-2681	433.84	0.756
	Survey Panel: weekly	“Has ATM / Debit card”	0.027	0.04	0.03	0.04	0.596	0.44-0.74	0.063	0.685
		“Often pays things in cash”	-0.029	0.035	-0.017	0.035	0.528	0.4-0.67	0.049	0.629
		“Has a credit card”	0.004	0.037	-0.054	0.036	0.488	0.32-0.61	0.052	0.334
		“Is uncomfortable trusting money to a bank”	-0.002	0.03	-0.028	0.03	0.23	0.13-0.38	0.041	0.641
		“Has vehicle or property insurance”	0.034	0.037	-0.027	0.037	0.645	0.51-0.78	0.052	0.506
		“Is not good at saving money”	-0.005	0.032	-0.002	0.032	0.327	0.2-0.44	0.045	0.984
		“Has savings account”	-0.05	0.034	0.00	0.034	0.272	0.16-0.41	0.048	0.362
	Google searching behavior: weekly	“Banco”	-5.565	3.691	5.62	3.72	71.755	53-100	10.75	0.086
		“Como abrir una cuenta”	1.685	8.874	-3.85	8.85	56.696	32-91	12.93	0.886
		“Robo”	-1.045	4.56	-3.67	4.55	77.02	63-100	6.72	0.715
		“BB&T”	-0.22	3.252	0.16	3.18	81.843	72-100	4.77	0.995
		“Bank”	-0.771	2.413	-0.79	2.414	92.304	79-100	3.77	0.908

Register to vote	Administrative: daily (log)	Voter registration by Hispanics, 01-01-2010 to 06-29-2013	0.062	0.35	0.006	0.35	7.378	0-11.64	1.504	0.976
	Administrative: weekly (log)	Voter registration by Hispanics, 01-01-2010 to 06-29-2013	0.102	0.34	0.04	0.34	9.626	6.27-12.13	1.032	0.950
	Survey Panel: weekly	"It is important to be informed to vote"	-0.007	0.036	-0.014	0.035	0.772	0.66-0.88	0.042	0.921
		"Reports being a member of any club / organization listed"	-0.01	0.038	0.015	0.039	0.327	0.2-0.44	0.047	0.898
		"Reports being registered to vote"	0	0.048	0.038	0.048	0.536	0.35-0.67	0.059	0.711
	Google searching behavior: weekly	"Rock the vote"	1.82	9.55	0.582	9.75	9.569	0-100	15.37	0.967
		"Eleccion"	-1.327	11.56	-3.45	11.79	10.167	3-100	13.571	0.917
		"Voto"	-0.916	10.93	-2.42	11.16	14.578	5-100	14.532	0.954
		"Votar"	-5.586	10.72	7.373	10.95	16.608	4-100	14.358	0.779
Don't drink and drive	Administrative: daily	Drunk driving arrests of Hispanics in 7 US cities	-0.817	4.64	3.52	4.63	27.315	1-72	13.93	0.737
	Administrative: weekly	Drunk driving arrests of Hispanics in 7 US cities	-14.37	14.80	-19.93	14.84	191.12	119-272	24.31	0.266
	Google searching behavior: weekly	"DUI"	0.274	4.61	-2.02	4.60	68.58	56-96	6.83	0.904
		"Borracho"	-1.807	5.05	5.28	5.05	67.951	45-100	12.83	0.520
Seek out university scholarships	Administrative: daily	Hits to Scholarship site recommended in the plotline	404.809	704.39	297.08	704.37	3232.8	0-22958	4315.58	0.776
	Survey Panel: weekly	"Owns 529 college savings"	0.008	0.007	-0.001	0.007	0.011	0-0.04	0.009	0.415
		"Owns other college savings"	0.013	0.008	-0.011	0.01	0.009	0-0.03	0.008	0.227
	Google searching behavior: weekly	"Yale"	4.022	3.90	-2.27	3.88	60.608	49-100	7.548	0.589
		"Beca"	-2.617	6.11	-1.40	6.14	51.873	24-91	14.95	0.754
		"Universidad"	-1.707	4.79	-2.23	4.81	79.137	56-100	8.341	0.641
		"Prestamo"	-7.512	8.88	-2.47	8.95	55.216	24-100	14.304	0.422
		"Diseño gráfico"	-6.313	10.09	-10.99	10.18	53.941	28-98	13.802	0.151
Eat healthy: Vegetables	Survey Panel: weekly	"Agrees that works on eating a well balanced diet"	-0.027	0.049	-0.036	0.049	0.555	0.41-0.69	0.049	0.660
		"Bought low-calorie foods when watching diet"	0.087	0.032	0.027	0.032	0.153	0.08-0.25	0.032	0.017
		"Agrees that eats food regardless of calories"	0.041	0.047	0.005	0.047	0.498	0.36-0.61	0.046	0.676
		"Presently watching diet"	0.01	0.045	0.09	0.045	0.457	0.34-0.58	0.045	0.138
		"Agrees that tries to eat healthy food / balanced diet"	-0.027	0.049	-0.036	0.049	0.555	0.41-0.69	0.049	0.660

		"Agrees that tries to include more fiber into diet"	0.002	0.05	-0.051	0.05	0.644	0.48-0.76	0.049	0.587
		"Agrees that looks for freshest ingredients when cooking"	0.03	0.046	0.04	0.05	0.594	0.48-0.73	0.045	0.557
		"Uses fresh produce"	0.007	0.032	0.03	0.032	0.919	0.81-0.98	0.031	0.614
		"Agrees that tries to eat healthier foods these days"	0.071	0.054	-0.005	0.054	0.605	0.42-0.76	0.054	0.430
		"Uses bagged/ packaged salad"	0.021	0.061	-0.037	0.061	0.659	0.46-0.8	0.059	0.775
		"Eats frozen vegetables"	0.027	0.064	-0.015	0.063	0.662	0.5-0.82	0.064	0.884
	Google searching behavior: weekly	"Papas fritas"	2.10	16.04	-2.33	15.997	61.912	34-100	16.161	0.980
		"Sobrepeso"	-10.095	16.83	1.72	16.74	48.559	0-100	22.14	0.829
		"Uvas"	2.776	7.28	-2.69	7.23	36.725	19-58	8.60	0.862
		"Mangos"	8.184	8.24	-18.08	8.26	71.176	36-100	16.165	0.056
		"Leche"	2.339	7.134	-3.71	7.11	80.118	53-100	7.399	0.821
		"Manzanas"	-4.827	10.19	8.62	10.20	51.373	27-100	14.397	0.616
		"Verduras"	4.944	11.002	9.31	10.99	58.059	0-97	15.34	0.642
Don't smoke	***Not implemented*	NA	NA	NA	NA	NA	NA	NA	NA	NA
Use carseats for infants	Survey Panel: weekly	"Car safety features are important"	-0.004	0.021	-0.09	0.022	0.67	0.55-0.75	0.031	0.0002
		"Looked up car safety rating before purchase"	-0.005	0.036	-0.005	0.036	0.45	0.3-0.58	0.05	0.982
		"Regularly wears seatbelt"	-0.01	0.037	0.001	0.04	0.69	0.51-0.81	0.05	0.921
	Google searching behavior: weekly	"Accidente automovilistico"	-7.658	13.52	-7.658	13.52	30.265	0-100	30.64	0.754
		"Carseat"	1.073	3.12	-3.61	3.11	74.392	58-100	7.012	0.463
