# Supplementary material for: Does Product Placement Change Television Viewers’ Social Behavior?
Source: PLoS One. 2015 Sep 23;10(9):e0138610. doi: 10.1371/journal.pone.0138610 (PMC4580471; doi:10.1371/journal.pone.0138610)
Supplement: S4 Table — This analysis uses the five-week period, the directly-randomized treatment unit, as the predictor of whether Googling about the topics of the broadcast message increased during that time period. The dependent measure is a standardized Google trends score per week, for “aceite de oliva,” “banco,” “voto,” “DUI,” “beca,” “verduras,” and “carseat.” We include fixed effects controls for each period, a control variable for week, calculate standard errors clustered by treatment unit. (PDF) [file pone.0138610.s004.pdf]

	Estimate	Std. Error	t value	Pr(>|t|)
(Intercept)	-0.64	1.03	-0.62	0.54
Five-week randomized period	-0.13	0.11	-1.17	0.24
Period 2	-0.29	0.19	-1.47	0.14
Period 3	-0.39	0.19	-2.05	0.04
Period 4	-0.43	0.27	-1.60	0.11
Period 5	0.58	0.29	1.99	0.05
Period 6	0.55	0.33	1.66	0.10
Period 7	1.02	0.41	2.46	0.02
Week of data	0.01	0.02	0.86	0.39
