# Supplementary figures and images for: Does Product Placement Change Television Viewers’ Social Behavior?
Source: PLoS One. 2015 Sep 23;10(9):e0138610. doi: 10.1371/journal.pone.0138610 (PMC4580471; doi:10.1371/journal.pone.0138610)

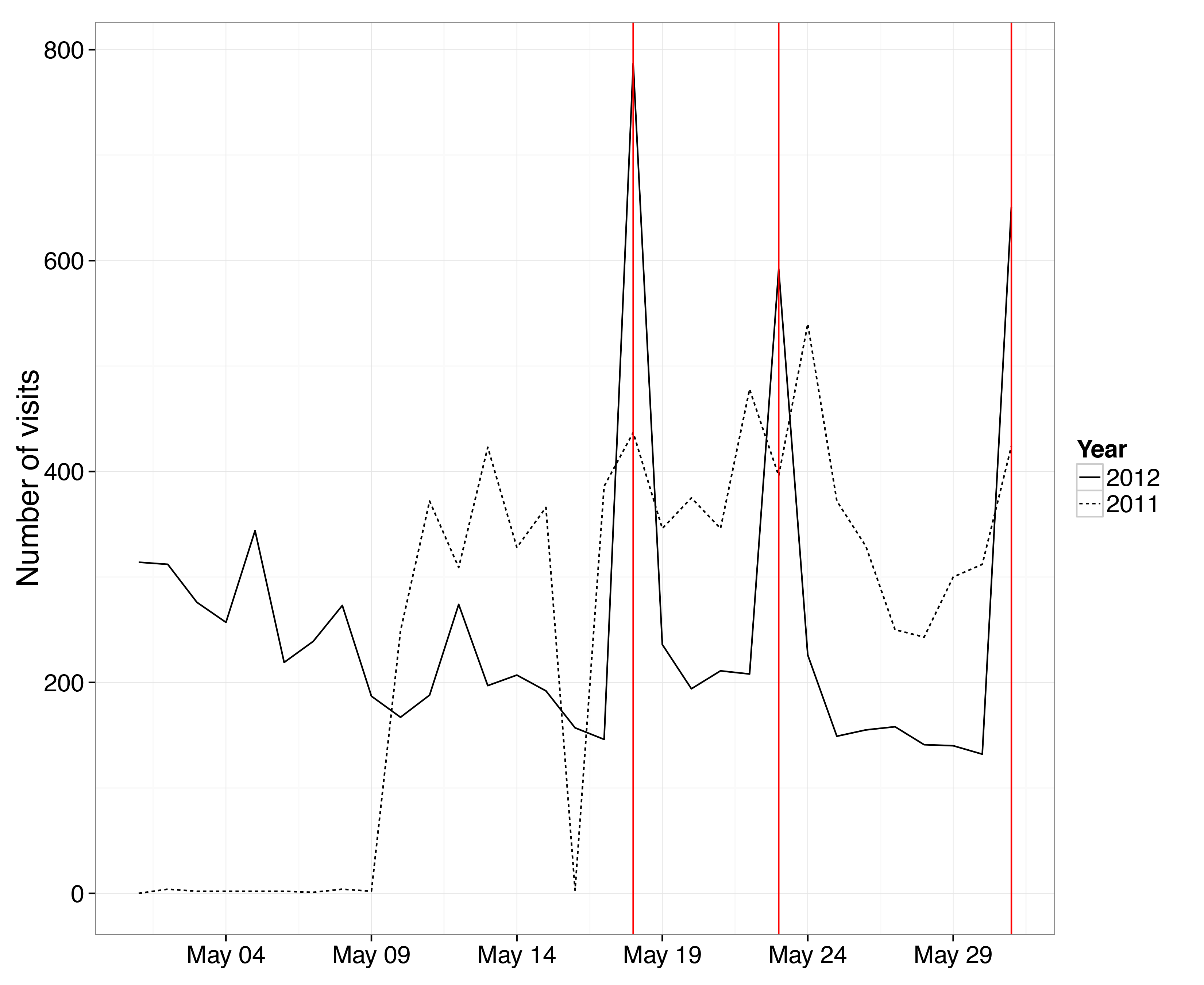

Supplement: S1 Fig — The solid line tracks the absolute number of all visits to the website in a month-long period before, during, and after the scholarship product placement scenes, which are indicated by the vertical lines. Unique visits to the website spike on each day that the scholarship message is broadcast on the telenovela. No such spikes in visitor numbers are observed on these days in the previous year, indicated by the dashed line. (TIF) [file pone.0138610.s005.tif]

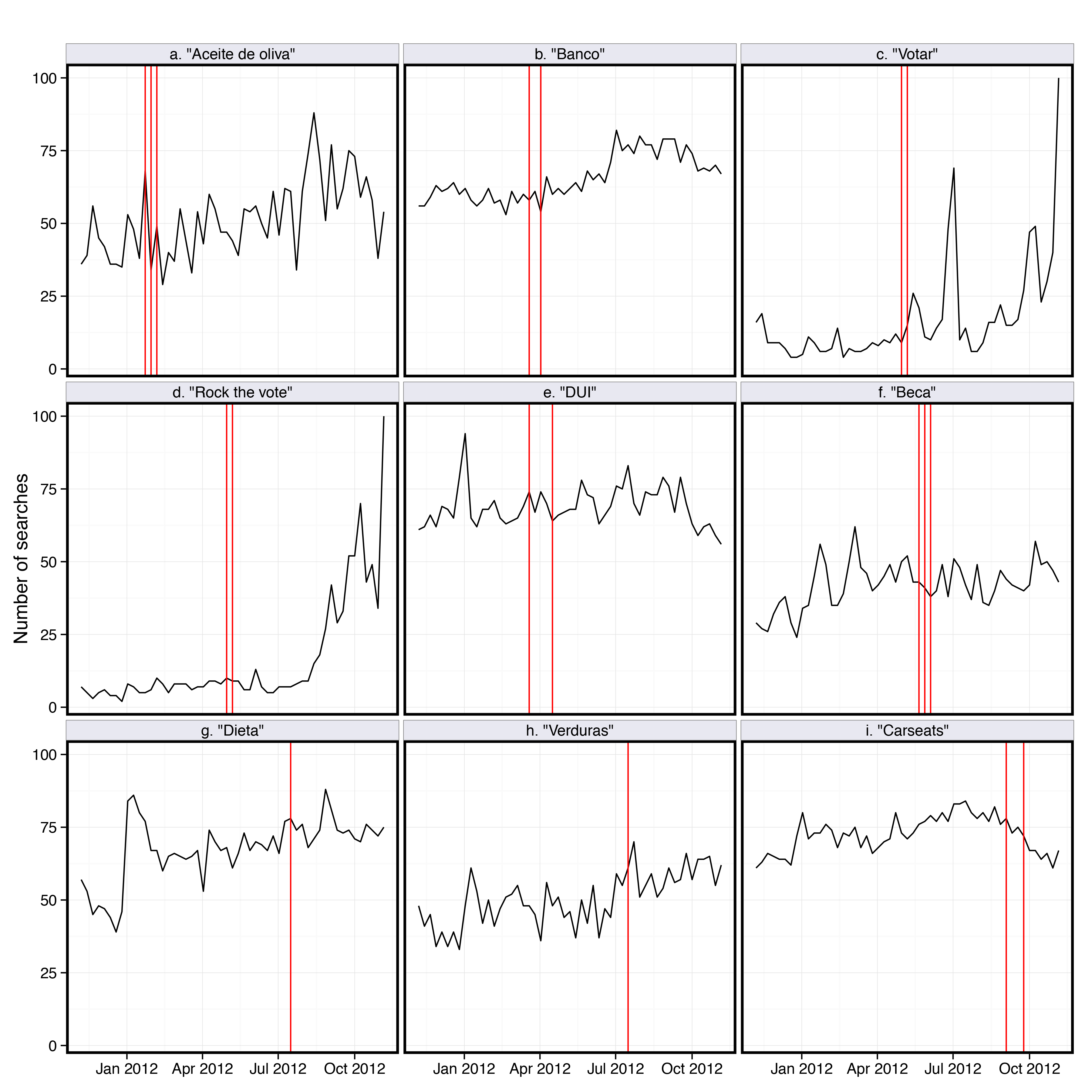

Supplement: S2 Fig — Nine figures, related to nine different messages, demonstrate that there is no detectable effect of the product placement into scenes broadcast during randomly assigned five week periods of the telenovela (the broadcast of the product placement is indicated by vertical lines). (TIF) [file pone.0138610.s006.tif]

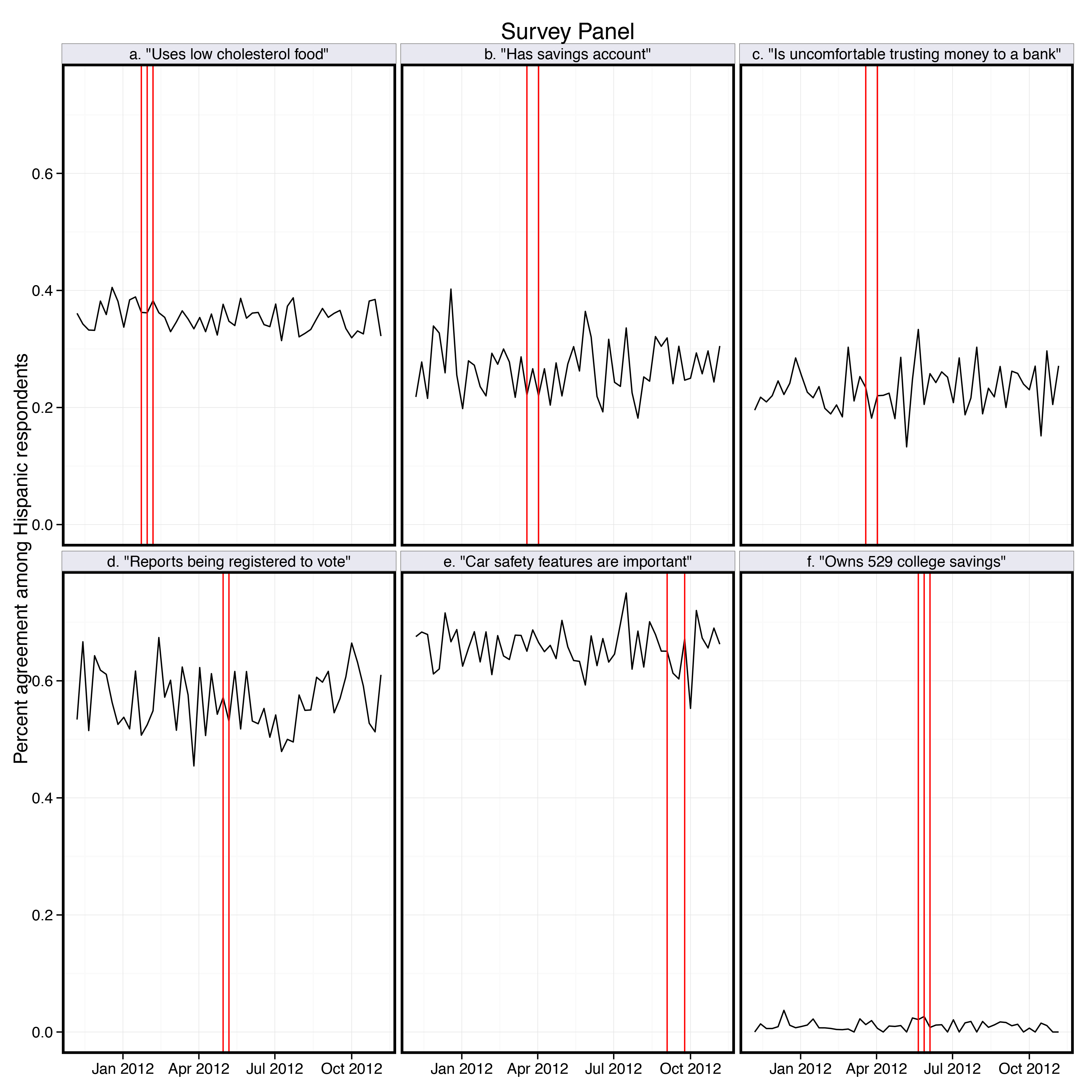

Supplement: S3 Fig — Six figures, related to six different messages, demonstrate that there is no detectable effect of the product placement into scenes broadcast during randomly assigned five week periods of the telenovela (the broadcast of the product placement is indicated by vertical lines). (TIF) [file pone.0138610.s007.tif]

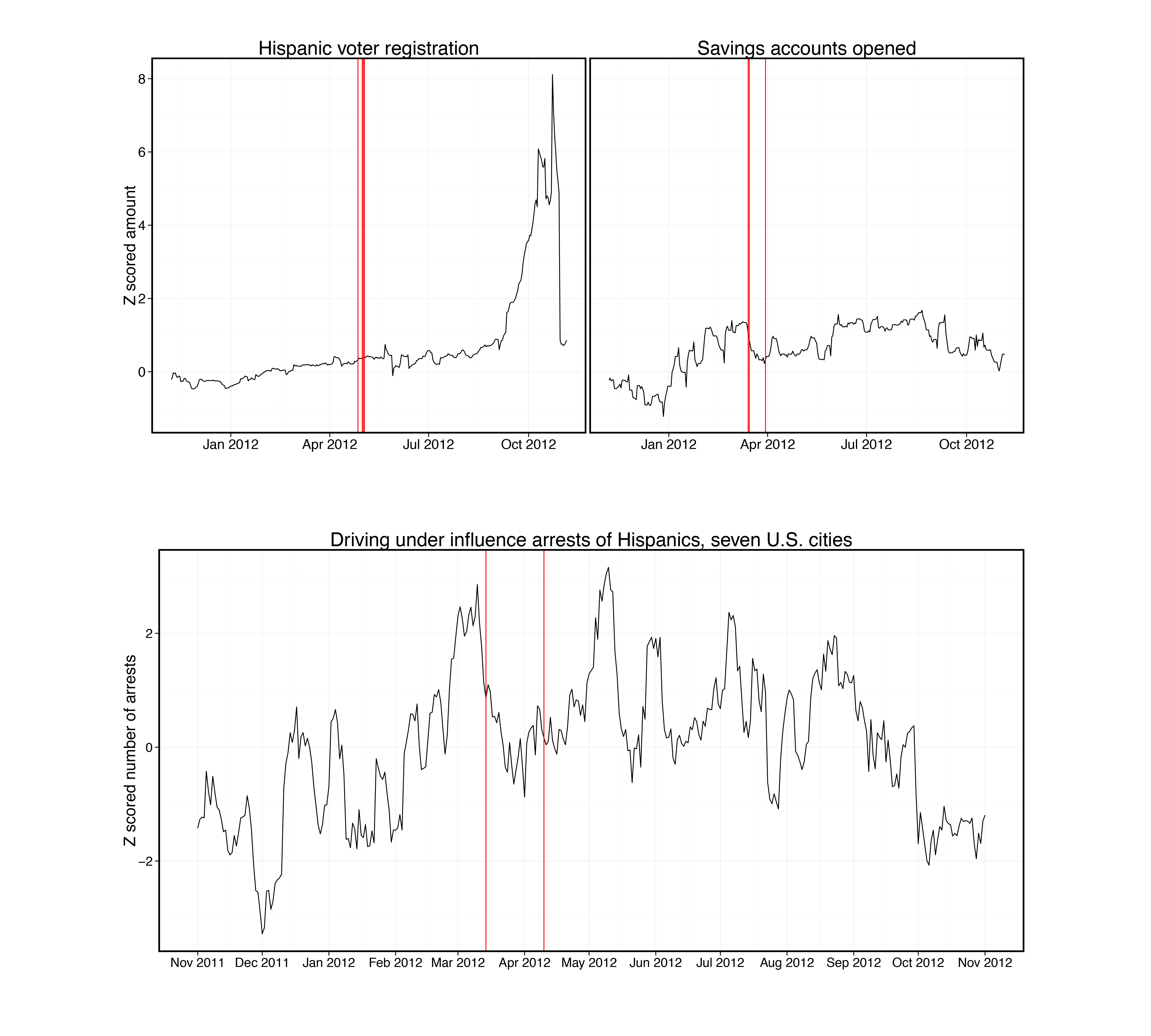

Supplement: S4 Fig — The top figure depicts numbers of arrests of Hispanic individuals for driving under the influence, z-scored and averaged across 7 major U.S. cities, over time. The occurrence of scenes depicting a “Don’t drink and drive” message is represented by the figure’s vertical lines. The bottom two figures show number of Hispanics who registered to vote over time, and the number of savings accounts opened at Hispanic consumer base branches of the bank that was featured in the telenovela. Those two figures test the efficacy of the “Register to vote” and “Open a bank account” messages, inserted into the randomly assigned broadcast periods as indicated by vertical lines. All three figures show negligible effects of the product placement on these behaviors. (TIF) [file pone.0138610.s008.tif]
