# Supplementary material for: Does Product Placement Change Television Viewers’ Social Behavior?
Source: PLoS One. 2015 Sep 23;10(9):e0138610. doi: 10.1371/journal.pone.0138610 (PMC4580471; doi:10.1371/journal.pone.0138610)
Supplement: S1 Text — (PDF) [file pone.0138610.s009.pdf]

2 Description of messages

Selection of messages for the Telenovela broadcasts: rationale, background on behaviors recommended, and description of messages in telenovela plots.

Issues of interest to Hispanics merit being studied. Today, the Hispanic population in the United States is calculated at 53 million.[1] This particular ethnic group encompasses approximately 17 percent of the country's population,[2] and around 14.2 percent of the nation's workforce.[3]

The size of the Hispanic population has more than doubled since 1990,[4] and is expected to continue to grow in the coming decades.[5] Given these stated facts, it should serve as a surprise that Hispanics are an understudied group.[6] Based on health, financial, educational, and even political indicators, we also find that many Hispanics are in a position wherein they could benefit from targeted and constructive information. With this in mind, our research project involved collaborating with scriptwriters in order to embed a series of messages in three Spanish-language telenovelas.

The first telenovela (from this point forward, Novela 1) is an adaptation of a 2002 Hollywood movie. The plot follows a Mexican immigrant and single mother living in New York City. While working as a maid in an upscale hotel, she begins a romantic relationship with the millionaire son of a U.S. senator. The show's initial episode aired in the fall of 2011 at 8:00 PM EST. Close to 1.6 million viewers saw the premier, and as the show progressed it captured the attention of approximately 1.53 million viewers per night.[7] Novela 1 ran for a total of 168 episodes.

The second telenovela (Novela 2) is based on a popular television series from Spain about the relationship between adolescents, parents, and teachers. The main storyline centers around a forbidden love between a high school teacher and her underage student. The show's first episode aired in the winter of 2012 at 10:00 PM EST. A cumulative audience of nearly 1.9 million viewers watched the premiere. In the city of Miami, the premiere was the most popular television show across all broadcast stations, regardless of language, for adults 18-49. Novela 2 ran for 108 episodes.

The third telenovela (Novela 3) follows the lives of two women. Both were childhood friends from differing socioeconomic classes who, because of a tragic experience in Mexico, are forced to separate. Years later, in Los Angeles, the two reunite and start working as bodyguards. The novela is filled with action scenes, and side stories that focus on various romantic relationships. Novela 3 began airing in March of 2012 at 9:00 PM EST. A cumulative audience of approximately 1.7 million viewers witnessed the premiere.[8] Given its popularity, the show aired for a total of 206 episodes.

The three novelas carried the positive messages that, according to various reports, could be of service to the Hispanic community. Summary information about the airing of each of the messages is presented in Table 1 in the paper. The first and the seventh message in our study's design suggested ways to lower the body's cholesterol and adopt a healthier diet. Compared to their White and African American counterparts, a greater percentage of Hispanic adult males have high cholesterol.[9] Obesity, a related medical condition, also threatens the Hispanic community.[10]

Thus, in collaboration with the network we embedded seven scenes in Novela 1 dedicated to the lower cholesterol message, and one scene in Novela 3 centering on the issue of healthy eating. As an example of one of those scenes, we see the novela's hero, a young and successful businessman, reacting to his father's recent heart attack by conducting Internet research on health-related questions. He looks concerned and focused as he types in the term, "bajar el colesterol" (in English, "lower cholesterol"). The character clicks on a website entitled, "Bajando el colesterol" (in English, "lowering cholesterol"). Viewers see the young businessman studying the website when his

[1] U.S. Census Bureau. "Asians Fastest-Growing Race or Ethnic Group in 2012". Washington, D.C., 2013. November 23 2013. <http://www.census.gov/newsroom/press-releases/2013/cb13-112.html%3E

[2] Ennis, Sharon R., Merarys Ros-Vargas, and Nora G. Albert. The Hispanic Population: 2010: United States Census Bureau, 2011.

[3] Grow, Brian. "Hispanic Nation." BusinessWeek March 15. 2004: 11.; Hochberg, Adam. "Drinking and Driving Plagues Latino Immigrants". Chapel Hill, North Carolina, 2006. U.S. News. NPR. November 29 2013; Kochhar, Rakesh. Latino Labor Report, 2008: Construction Reverses Job Growth for Latinos. Washington, D.C., Pew Hispanic Center.

[4] Owens, Anna M. "Hispanics in the United States". 2006. Ed. US Census Bureau. Ethnicity and Ancestry Branch, Population Division, US Census Bureau. November 18 2013.

[5] NCLR. "20 Faqs About Hispanics". Online, 2014. National Council of La Raza. September 17, 2014 2014.

[6] de la Garza, Rodolfo O. "Latino Politics." Annual Review of Political Science 7.1 (2004): 91-123; Dovidio, John F., et al. "Understanding Bias toward Latinos: Discrimination, Dimensions of Difference, and Experience of Exclusion." Journal of Social Issues 66.1 (2010): 59-78.

[7] Anonymous. "Broadcast Tv Ratings Watch". Coral Gables, FL, 2012. Hispanic Market Weekly. July 4 2012.

[8] TVboricua.USA. "Ratings Telenovelas USA". 2012. July 12 2012.

[9] U.S. Department of Health and Human Services. Health, United States, 2012: With Special Feature on Emergency Care. Hyattsville, MD: Center for Disease Control and Prevention, 2013.

[10] CDC. "Compared with Whites, Blacks Had 51% Higher and Hispanics Had 21% Higher Obesity Rates". 2010. Center for Disease Control and Prevention. November 24 2013.; Let's Move! "Learn the Facts". 2010. November 24 2013.

assistant walks into the office. The two engage in a twenty-second sobering conversation about the severe health risks posed by cholesterol. The scene ends with each asking the other whether he has had his cholesterol levels checked recently.

From a financial perspective, many Hispanics remain disconnected from the banking system. In fact, approximately half of the country's Hispanic population does not own a savings or checking account.[11] This represents an obstacle to proper financial management and wealth building. Being unbanked also leads to considerable safety risks,[12] which is an issue that we highlight across three scenes in Novela 1. The first of those scenes opens with the novela's main character and her family returning home only to find that their modest apartment has been broken into. The main character's mother discovers that her savings, which she had stored in the kitchen, have been stolen. The victim is, thus, encouraged time and time again by primary and secondary characters in the novela to open a savings account. Twelve episodes later, the victim arrives at a bank. A courteous bank agent welcomes her in Spanish and they engage in an informative conversation. Based on this exchange, we learn that banks are not just institutions for the wealthy. We also learn that, thanks to checkbooks and debit cards, bank customers always maintain direct access to their funds. Finally, the bank agent goes through the list of documents that one needs to present if he or she does not own a Social Security Number. After hearing all this, the novela character happily exclaims: "Well, then, let's open the account! Here are my documents" (authors' translation).

In the realm of politics, Hispanics are punching below their weight. Over the past three presidential elections voter turnout rate has increased in a consistent manner for Hispanics.[13] Still, even as Hispanics represent 17% of the U.S. population, they were only 10% of the electorate in the 2012 elections. Furthermore, of the 23.7 million eligible Hispanic voters, only about half cast a vote.[14] Thus, in an effort to encourage Hispanic viewers to register to vote, in Novela 1 we embedded six scenes spread across four episodes promoting political engagement. In one of those scenes we see one of the secondary characters type in "ROCK THE VOTE" on an Internet search engine. In yet another we see the same character walking the streets of Queens, NY. He is helping the novela's hero record a new commercial to encourage Hispanics to register to vote. "This is my barrio," we hear the character say in Spanish, "a place where a number of Hispanics live and work. Today, I'm very excited; I was just granted US citizenship. And do you know what was the first thing I did? I registered to vote. I too want to play a role in this democracy." The camera continues to roll when the novela's hero appears on the scene and invites people to help strengthen the Hispanic vote.

Same as in politics, Hispanics are underrepresented in higher education. According to the Pew Hispanic Center, young Hispanics are less likely than their White counterparts to enroll in a four-year college (56% versus 72%). Similarly, they are less likely to be enrolled in college full time and less likely to complete a bachelor's degree.[15] Lack of access to financial aid and lack of awareness of financial aid opportunities are an important part of the problem.[16] Thus, in light of this issue, we included three scenes in Novela 3 encouraging young viewers to apply to college and to seek financial aid through the Hispanic Scholarship Fund's website. In one of those scenes we see the novela's main character walking into his sister's room. She is sitting at her desk with a laptop. She explains her frustration at not being able to find proper information about college scholarships. He, in turn, offers her a few words of encouragement and then suggests that she look at the same website he had used when applying to college: www.tuspalabrasdehoy.org. We see her write this address into a search engine, and then we see the Hispanic Scholarship Fund's website appear on the screen. "There it is," the main character says in Spanish, "all the information you need." He adds, "Where do you think I got the information for my scholarship?"

According to the National Highway Traffic Safety Administration, motor vehicle crashes are the leading cause of death for Hispanics under the age of 34.[17] This is explained by a number of factors. For one, the use of child car seats (or similar safety mechanisms) by Hispanics is significantly lower than non-Hispanics.[18] In other words, Hispanic children face, on average, a higher risk of death or injury while riding in an automobile. It is also the case that, nationwide, drunk driving is a considerable problem among Hispanics. In fact, 47% of Hispanic crash deaths are alcohol-related, compared with 40% for the general population. Hispanics rank second only to Native Americans in their alcohol death rate on the highway.[19] Thus, in light of these issues, in Novela 2 we promoted the idea that choosing not to drive when under the influence of alcohol is the manly thing to do, and in Novela 3 we encouraged parents to secure their children to an infant car seat before driving. Twice characters in the novelas communicated each of the two messages in two distinct episodes.

---

[11] Muiz, Brenda, et al. Financial Education in Latino Communities: An Analysis of Programs, Products, and Results/Effects. Washington, D.C.: National Council of La Raza, 2004.

[12] Bass, Paul. "Immigrants' Wake". New Haven, CT, 2006. New Haven Independent. September 2014.; Lagunes, Paul, Brian Levin, and Ruth Ditlmann. "Documenting the Undocumented: A Review of the United States' First Municipal Id Program." Harvard Journal of Hispanic Policy 24 (2012).

[13] Lopez, Mark Hugo, and Paul Taylor. Dissecting the 2008 Electorate: Most Diverse in U.S. History. Washington, D.C.: Pew Research Center, 2009.

[14] Taylor, Paul, et al. An Awakened Giant: The Hispanic Electorate Is Lilely to Double by 2030. Washington, D.C.: Pew Research Center: Pew Hispanic Center, 2012.

[15] Fry, Richard, and Paul Taylor. Hispanic High School Graduates Pass Whites in Rate of College Enrollment: High School Drop-out Rate at Record Low. Washington, D.C.: Pew Hispanic Center, 2013.

[16] HSF. Revelations and Recommendations: The 2000 Hsf Hispanic Education Study. Gardena, CA: Hispanic Scholarship Fund, 2001; "About Hsf". Gardena, CA, 2014. Hispanic Scholarship Fund. September 23 2014. <http://hsf.net/en/about-hsf/%3E.

[17] Copeland, Larry. "Anti-Drunken-Driving Efforts Aimed at Latinos". 2007. News: Nation. USA Today. November 29, 2013 2007. <http://usatoday30.usatoday.com/news/nation/2007-04-10-hispanic-dui_N.htm%3E.

[18] Pickrell, Timothy M., and Tony Jianquiang Ye. The 2009 National Survey of the Use of Booster Seats. Washington, D.C.: U.S. Department of Transportation: National Highway Traffic Safety Administration, 2010.

[19] Hochberg, Adam. "Drinking and Driving Plagues Latino Immigrants". Chapel Hill, North Carolina, 2006. U.S. News. NPR. November 29 2013. <http://www.npr.org/templates/story/story.php?storyId=5572465%3E.

In order to gauge the messages' effects we collected outcome data that, directly or indirectly, measured shifts in behavior and in self-reported behavior.

For the banking message we collaborated with BB&T, a Fortune 500 company that owns nearly two thousand financial centers in over ten states and Washington, D.C. This particular bank shared a dataset indicating the number of checking accounts opened across each of its financial centers between November 29, 2010 and May 3, 2013. The dataset distinguished between regular bank branches and those that have Spanish-speaking tellers (which indicated a relatively sizable Hispanic customer base).

In order to gauge the effect of the register to vote message, Rock the Vote made available their daily website hits information for their Spanish-language website (<http://www.rockthevote.com/en-espanol/>) between Monday, February 22, 2010 and Wednesday, July 31, 2013.

For the scholarship message we sought the help of the Hispanic Scholarship Fund (or HSF). Founded in 1975, this is the largest nonprofit supporting higher education among Hispanics.[20] HSF shared their hourly and daily website hits information for their <www.tuspalabrasdehoy.org>, a website that provides facts about scholarship programs and means for applying to said programs.

We used Google Trends to assess whether the use of relevant search terms increased after each of the different messages aired. Google Trends data provides indexed values of the relative frequency with which people search Google for a particular term. Kearney and Levine,[21] for example, have used this sort of data as proxies that "provide some gauge of what viewers are thinking about when they watch the show." The terms we tested were either mentioned explicitly in the novelas as part of a message (e.g., "egg white substitutes" and "DUI"), or, even if they were not part of the novela's script, were strongly associated with one of the messages (e.g., "carseat"). Some terms were not popular enough to register on Google Trends. An example of search a term is "prueba del colesterol" (in English, "cholesterol exam").

To measure self-reported outcomes, we obtained panel survey data from Experian, a consumer data company. The data are via the Experian-Simmons National Consumer Study, which is a continuously fielded survey of approximately 25,000 U.S. adults, including both English and Spanish speakers, on a weekly basis. The survey covers lifestyles, attitudes, brand preferences, and media use. We obtained data from January 2008 through December 2012. The survey uses a two-phase data collection method: A telephone placement interview or mail-based recruitment questionnaire to obtain the household's participation in the survey and then the mailing of self-administered survey booklets to eligible household members. Respondents are offered upfront cash incentives or sweepstakes. Data were delivered as moving estimates for a defined population, not individuals. The populations we tracked are: all adults, respondents who identify as Hispanic or Latino/a, who do not identify as Hispanic or Latino/a, who are Spanish-dominant (i.e., speak mostly Spanish or only Spanish at home), who are Spanish-language novela viewers, and who are viewers of novelas on the Spanish-language broadcasting company we worked with. Results reported in the paper are for adults who identify as Hispanic or Latino/a, the most relevant sample frame, but our results do not change when we narrow the population pool to those who view novelas.

The Experian Hispanic sample is fairly representative of the Hispanic population in the U.S., although it is different from the audience we targeted, as assessed by the broadcast network, on some select characteristics: 55% of the Experian sample is foreign-born, compared to 88% of the broadcast network audience, and 43% of the Experian sample has a household income below $40,000, compared to 63% of the broadcast network audience.

[20] HSF. Revelations and Recommendations: The 2000 Hsf Hispanic Education Study. Gardena, CA: Hispanic Scholarship Fund, 2001. "About Hsf". Gardena, CA, 2014. Hispanic Scholarship Fund. September 23 2014. <http://hsf.net/en/about-hsf/%3E.

[21] Kearney, Melissa S., and Phillip B. Levine. "Media Influences on Social Outcomes: The Impact of Mtelevision's 16 and Pregnant on Teen Childbearing." The National Bureau of Economic Research2014. 54.
