# Supplementary material for: Does Product Placement Change Television Viewers’ Social Behavior?
Source: PLoS One. 2015 Sep 23;10(9):e0138610. doi: 10.1371/journal.pone.0138610 (PMC4580471; doi:10.1371/journal.pone.0138610)
Supplement: S2 Text — (PDF) [file pone.0138610.s010.pdf]

12 de septiembre del 2011

Primer mensaje prosocial para *Telenovela 1*:
Estrategias para sugerirle a los televidentes que reduzcan su colesterol

Capítulos y fechas entre las cuales se deberá presentar los mensajes sobre el colesterol:

	Inicio del periodo	Fin del periodo
**Día**	lunes, 16 de enero del 2012	viernes, 17 de febrero del 2012
**Capítulo**	36	60

Resumen informativo sobre el colesterol:

El colesterol es una sustancia grasosa que el cuerpo utiliza para proteger los nervios, formar tejidos celulares, y producir ciertas hormonas. A pesar de que algo de colesterol es necesario, el exceso de colesterol en la sangre se puede almacenar en las arterias. La acumulación de colesterol dentro de las arterias se conoce con el nombre de "placa". Tal y como lo muestra la siguiente imagen, la placa hará que las arterias se angosten y endurezcan.

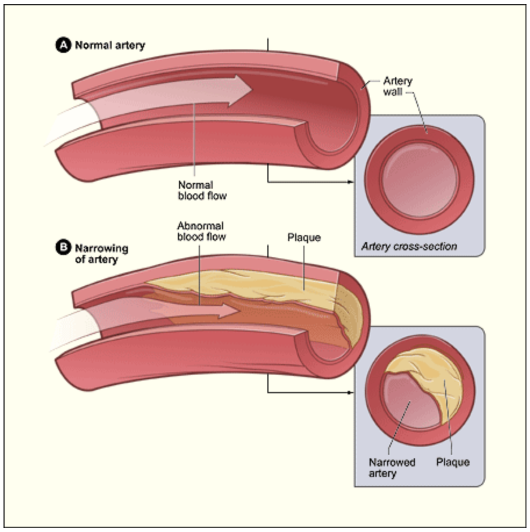

Los depósitos grandes de colesterol pueden bloquear por completo una arteria. Las placas de colesterol también se pueden rajar causando la formación de un coágulo que bloquea el flujo de la sangre. Si una arteria que suministra sangre a los músculos de su corazón se bloquea esto puede causar un ataque al corazón (también conocido como "infarto").

Entre la población Latina/Hispana en los Estados Unidos se estima que un 34.1% sufre de niveles altos de colesterol. Esto ayuda a explicar el número elevado de muertes entre la población Latina/Hispana a causa de problemas del corazón. Tomando en cuenta este problema queremos recomendar que la novela incluya mensajes prosociales que nos permitirían medir mejoras en los hábitos de la población Latina/Hispana en los Estados Unidos. Los hábitos que podríamos medir son: las búsquedas de información que realizan los televidentes a través de Google; la compra de comida saludable en los supermercados; y los hábitos de ejercicio y la compra de equipo de ejercicio.

La problemática:

Conforme a lo discutido en la reunión del 16 de agosto entre Paul Lagunes y el guionista, el político dentro de *Telenovela 1* podría sentir un dolor intenso en su pecho. Esto no sería cosa menor. Sus asesores más cercanos se preocuparían ya que se podría desatar un escándalo si la gente se enterara de que él hombre no está bien de salud. Su esposa también se preocuparía.

El político iría al doctor para que le hicieran un examen médico. Los resultados mostrarían que las arterias del político presentan una acumulación de colesterol. El doctor le explicaría al político que si no come sanamente y si no hace ejercicio, podría hasta sufrir de un ataque al corazón.

Las soluciones:

A continuación presentamos tres posibles métodos para ayudar a combatir el colesterol. Les solicitamos de la forma más atenta que por favor incluyan los tres métodos dentro de la novela, ya que así podríamos lograr un mayo impacto prosocial sobre la audiencia.

1) **El primer método sobre la necesidad de informarse más sobre el problema del colesterol:**
   Como sugerencia, se podría mostrar como uno (o varios) de los personajes intenta (o intentan) obtener más información sobre el tema del colesterol realizando una búsqueda en Google usando las palabras “alto colesterol”.

   Esta sugerencia se basa en que descubrimos que Google cuenta con una herramienta que contabiliza las búsquedas que realiza la gente. Por lo mismo, sugerimos que diferentes personajes dentro de la novela muestren curiosidad por el tema e intenten realizar una búsqueda en Google usando las palabras “alto colesterol”. Así podremos medir si hubo más gente investigando este tema de salud tan importante gracias a la novela.

   Los personas podrían realizar su búsqueda en Internet dado el susto que les causa los problemas de salud del político:

- Algunos de los asesores del político y algunos ciudadanos podrían ingresan las palabras “alto colesterol” en Google para realizar su búsqueda en Internet.
- La esposa del político podría también ingresar las palabras “alto colesterol” en Google para realizar su búsqueda en Internet.

Muy importante: por favor muestren a los personajes realizando sus búsquedas en Internet usando el servicio de Google (o algo que de la apariencia de que se está usando Google) e ingresando las palabras “alto colesterol”.

2) **El segundo método sobre las ventajas de cocinar usando ingredientes más sanos:**
El colesterol puede provenir directamente de la comida que uno ingiere. Por lo mismo, para mejorar sus niveles de colesterol una persona también debe mejorar sus hábitos alimenticios sin necesariamente dejar de disfrutar el sabor de lo que come. Muchos televidentes no van a saber qué alimentos deben o no ingerir, por lo que la novela les podría presentar dos opciones interesantes: uno que tenga que ver con el uso de claras de huevo y el otro que tenga que ver con el uso de aceite de oliva. Por ejemplo:
    - La esposa del político le podría preparar a un desayuno saludable. Dicho desayuno incluiría avena y huevos revueltos hechos exclusivamente con claras. Quizá el político se reusa a comer lo que la esposa le ofrece, por lo que ella y hasta sus asesores lo tratan se esfuerzan por convencerlo de que mejore su dieta. (Quizá hasta algún reportero podría tratar de influenciar los hábitos alimenticios del político, ya que su salud ya se ha vuelto noticia.)
    - El político y su esposa podrían ir a casa de unos amigos para cenar. En dicha escena los anfitriones les ofrecerían unos platillos tradicionales (por ejemplo, tamales, mole, arepas, etc.). Los invitados disfrutarían la comida e iniciarían una plática sobre la importancia de ingerir alimentos que presentan niveles bajos de colesterol. Durante la plática los hombres podrían hablar de lo tanto que disfrutan los platillos tradicionales que les preparaban sus madres y las mujeres podrían ofrecer ideas de cómo preparar esos mismos platillos usando ingredientes más sanos (por ejemplo, se podría usar aceite de oliva en vez de manteca). Haciéndose el difícil, el político podría mostrarse escéptico. Apuntando a la comida que tienen sobre la mesa podría decir algo así como, ”Los platillos tradicionales no saben tan ricos cuando se usan ingredientes sanos.” Pero en ese momento la anfitriona lo sorprendería con la noticia que todo se cocinó usando aceite de oliva en vez de manteca.

3) **El tercer método sobre la importancia de hacer ejercicio:**
El ejercicio puede reducir los niveles de colesterol. Por lo mismo, una sugerencia es que el político se podría comprar unos tenis nuevos con los

cuales ir a correr o caminar en algún parque. Uno de sus asesores hasta le podría comprar los tenis como regalo y le podría recomendar una trayectoria diaria que le permitiría caminar desde su hogar hasta su oficina, pasando por las casas de sus electores. El punto es que el político podría aprender que con tal solo adquirir y utilizar unos zapatos para caminar podría mejorar su salud.

21 de diciembre del 2011

Segundo mensaje prosocial para *Telenovela 1*:
Estrategias para sugerirle a los televidentes que abran una cuenta bancaria

Capítulos y fechas entre las cuales se presentarán los mensajes que “open a bank account:

	Inicio del periodo	Fin del periodo
**Día**	20 de febrero, 2012	26 de marzo, 2012
**Capítulo**	61	85

Sobre el problema actual:

El no contar con una cuenta bancaria conlleva riesgos muy importantes. El caso de Manuel Santiago, un Tlaxcalteca de 36 años que vivía en el estado de Connecticut como indocumentado, ejemplifica tales riesgos. Manuel fue asesinado a cuchilladas después de haber cobrado un cheque.[1] Lo más triste de esta situación es que Manuel se pudo haber salvado si hubiera contado con una cuenta bancaria. Es más, son muchos los hispanos/latinos en los Estados Unidos que estarían más a salvo guardando su dinero en un banco.

Al contar con una cuenta bancaria las empresas pueden realizar sus pagos salariales como depósitos directos. Ya no es necesario cargar con tanto efectivo, ni guardar el dinero bajo el colchón. También es cierto que, al contar con una cuenta, deja de ser necesario pagarle una comisión a las agencias que se dedican a cambiar cheques. En fin, el abrir una cuenta bancaria tiene sentido. Sin embargo, a pesar de los beneficios que conlleva ser cuentahabiente, sólo el 63 por ciento de los adultos latinos/hispanos en los Estados Unidos tienen una cuenta bancaria. Este porcentaje es bajo, sobre todo si lo comparamos con el porcentaje de adultos no-hispanos/latinos en el país que tienen una cuenta de banco (85 por ciento).

Hay varias razones por las que los latinos/hispanos no abren una cuenta . Quizá la principal es que muchos no confían en los bancos. Creen que son instituciones en los que no se habla español o en los que se les va a pedir un comprobante de ciudadanía para poder abrir una cuenta.

[1] Para más información ver: http://www.newhavenindependent.org/index.php/archives/entry/immigrants_wake/

El mensaje prosocial:

Dado todo lo anterior, se le sugerirá a los televidentes que abran una cuenta bancaria para así guardar su dinero de forma segura y sin tener que pagarle comisiones a un tercero. También se les informará de lo siguiente:

1. Uno no necesita de un "Social Security Number" o de un "Tax Payer ID" para abrir una cuenta bancaria.
2. Aunque uno no sea ciudadano estadounidense puede abrir una cuenta bancaria presentando dos documentos. Normalmente deben presentar un pasaporte (sea del país que sea) y un documento secundario. Como documento secundario puede presentar la matrícula consular, la tarjeta de elector mexicana (al que comunmente le dicen "IFE"), una licencia de manejo vigente (del país que sea), o una identificación proveida por una escuela o empresa.
3. Hoy en día, los bancos suelen contar con empleados que hablan en español.
4. Uno tiene el derecho de solicitar que se le expliquen las reglas que rigen las cuentas bancarias. Algunas cuentas, por ejemplo, requieren que el cuentahabiente siempre guarde una cantidad mínima.

Nota: la clave está en lograr que los televidentes <u>le pierdan el miedo</u> al sistema bancario en los Estados Unidos.

Posibles escenarios:

El mensaje tendrá un mayor impacto si se muestra como es que uno de los personajes hispanos/latinos visita un banco y, sin molestia alguna, logra abrir una cuenta de cheques. Durante su visita al banco sería importante que los empleados del banco le hablen al personaje en español y lo/la traten amablemente.

Se nos ocurre que, como parte de la historia, Tarek o uno de los socios del polaco le podrían robar su dinero a Goyo. Al descubrir que fue victimizado, Calixta, quien le sabe al mundo de los negocios, le podría ayudar a abrir una cuenta a su compañero de barrio para que así pueda guardar su dinero con mayor seguridad.

Quizá Amador le confía a Calixta que, durante años, ha guardado todo su dinero en una simple maleta (¡sí sucede!). Al ver esto, Calixta le podría ayudar a abrir una cuenta bancaria.

Otra posibilidad es que, en su afán por ayudar a su padre o a su madre y dada su inteligencia, Lalo asesora a Victor o a Marisa para que abran una cuenta.

Una opción adicional es que, como parte de la campaña política de Cristóbal, Tito propone que le ayuden a la gente de Queens a abrir cuentas bancarias.

Tercer y último mensaje prosocial para *Telenovela 1*:
Estrategias para sugerirle a los televidentes que se REGISTREN PARA VOTAR

Capítulos y fechas entre los cuales se presentarán los mensajes que invitan a los televidentes a REGISTRARSE PARA VOTAR:

	Inicio del periodo	Fin del periodo
**Día**[2]	27 de marzo, 2012	30 de abril, 2012
**Capítulo**	86	110

Voz y voto:

Es sorprendente ver cómo cambió el discurso político de los candidatos republicanos a la presidencia una vez que empezaron a competir por la nominación en Florida. Dado que el electorado de ese estado tiene rostro hispano, los políticos empezaron a producir anuncios en español, a hablar en favor de la inmigración, y en general a buscar atraer el voto de las personas de origen latinoamericano.

Lo que esto demuestra es que es importante promover el voto entre la población latina en los Estados Unidos, sobre todo si lo que se busca es promover los intereses de este grupo minoritario entre la clase política.

El mensaje prosocial:

Se le sugerirá a los televidentes que se registren para votar cumpliendo con todas las obligaciones que establece la ley. Se podría mostrar como uno o varios de los personajes intentan convencer a otros que se registren para votar. Además, se podría mostrar como uno (o varios) de los personajes intenta (o intentan) registrarse para votar visitando la página <http://www.rockthevote.com/en-espanol/>.

Posibles escenarios:

- Aunque algunos de los personajes en *Telenovela 1* no puedan votar por su calidad de inmigrantes indocumentados, quizá alguno de ellos podría obtener la ciudadanía antes de las elecciones y podría entonces buscar informarse sobre lo

[2] Nota: estas fechas están sujetas al calendario televisivo de Telemundo, mientras que los capítulos sí son parámetros fijos.

que debe hacer para registrarse para votar. Otra opción es que alguno de los personajes podría ser convencido de que su voto es necesario.

- Dado que muchos de los hijos de indocumentados sí tienen la ciudadanía estadounidense, quizá podría haber un episodio en el que Lalo le explica a sus compañeros de la escuela cómo le tendrán que hacer para que, después de haber cumplido los 18 años, puedan votar.

Requisitos para poder votar en los Estados Unidos:

1. Sólo los ciudadanos estadounidenses que cuenten con los 18 años de edad llegado el día de la elección pueden registrarse para votar.

2. Las personas pueden registrarse para votar a través del internet visitando la siguiente página: <http://www.rockthevote.com/en-espanol/>.

3. En algunos estados se necesita la licencia de manejo para poder votar.

4. Normalmente uno se debe registrar por lo menos dos semanas antes de la elección para poder votar.

Respuestas a las preguntas más comunes:

- ¿Es cierto que si me registro para votar el gobierno me puede emplazar para cumplir con mi "deber de jurado" (en inglés, *jury duty*)?
  En la mayoría de los estados de la unión americana, el poder judicial hace su selección en base al listado de personas con licencia para conducir y no en base al listado de personas que están registradas para votar.

- ¿No será que si me registro para votar el gobierno obtendrá acceso a mi información personal?
  Cualquier información que uses para registrarte para votar es información que muchos (incluyendo el gobierno) ya conocen sobre ti. De cualquier forma, el estado cuidará toda información que le proveas.

- Creo que me registré para votar hace varios años. ¿Seguiré registrado o me tengo que volver a registrar?
  Si ya no recibes folletos electorales por correo, te deberías volver a registrar para votar. La mayoría de los estados borran los nombres de aquellas personas que de forma consecutiva no votan en varias elecciones.

- ¿Mi voto es secreto?
  ¡Claro que sí! Tu voto en los Estados Unidos es secreto. Sólo tú sabrás a quien elegiste.

- ¿Para que voto si mi voto no hace la diferencia?
  ¡Claro que tu voto hace la diferencia! ¡Cada voto cuenta! Simplemente recuerda que durante las últimas elecciones presidenciales (por ejemplo, en la elección entre George W. Bush y Al Gore) los resultados han sido muy cerrados.

Primer mensaje prosocial para *Telenovela 2*:
Estrategias para sugerirle a los televidentes que no conduzcan bajo la influencia del alcohol

Capítulos y fechas entre las cuales se presentarán los mensajes que sugieren no conducir bajo la influencia del alcohol:

	Inicio del periodo	Fin del periodo
**Día**[3]	14 de marzo, 2012	17 de abril, 2012
**Capítulo**	36	60

Resumen informativo sobre los riesgos de conducir bajo la influencia del alcohol:

Los accidentes automovilísticos representan la quinta causa de muerte entre Hispanos/Latinos en los Estados Unidos. Este problema se debe, en gran parte, a que muchos conducen bajo la influencia del alcohol. Es más, entre los estadounidenses de descendencia mexicana, un quince por ciento ha sido multado por haber manejado en estado de ebriedad.

El límite de copas:[4]

La cantidad de alcohol que hay presente en el cuerpo de una persona es medida por el peso del alcohol en un volumen específico de sangre. A esta fórmula se le llama concentración de alcohol en la sangre o "BAC" (por sus siglas en inglés).

El alcohol disminuye la habilidad que tiene una persona para conducir un vehículo de forma segura. Un conductor con un nivel de concentración de alcohol en la sangre de 0.08 a 0.10 tiene 11 veces mayor riesgo de involucrarse en un choque que los conductores con un contenido de alcohol en la sangre de cero. Este mismo riesgo es 52 veces mayor entre los hombres jóvenes.

Las consecuencias legales de conducir bajo la influencia:

Todos los estados en los Estados Unidos han aprobado leyes que prohíben conducir con una concentración de alcohol en la sangre de 0.08 o mayor. Si a uno se le

[3] El cálculo de las fechas está basado en que el programa se estrenará el 24 de enero.
[4] Para más información ver: http://www.stopimpaireddriving.org/ABCsBACSpanWeb/page2.htm

encuentra conduciendo bajo la influencia del alcohol, se le puede arrestar. Dicho conductor también puede perder su licencia de manejo por un periodo de, por lo menos, 3 meses. Y si alguien hiere a otra persona al estar manejando bajo la influencia del alcohol, puede acabar en la cárcel por un periodo prolongado.

El mensaje:

Dado todo lo anterior, se le hará una recomendación muy importante a los televidentes: **pídele a un amigo o a una amiga sobrio(a) que te ayude a conducir si has estado tomando.**

La clave está en comunicar esta recomendación de tal manera que los hombres vean ejemplos de cómo pueden ceder las llaves de su auto sin perder el respeto de los demás o sin que su hombría quede afectada.

Recomendamos que sean los personajes masculinos quienes tengan que ceder sus llaves al buscar que alguien los apoye para conducir. Hay una razón muy importante detrás de esto. La mayoría (83%) de los Latinos/Hispanos que manejan bajo la influencia del alcohol son hombres.

Posible escenas:

Sugerimos que este mensaje **se presente más de una vez** para así lograr un mayor impacto sobre la audiencia. Es más, si es posible, pedimos que este mismo mensaje se presente en dos o tres capítulos.

Podría ser que el personaje está en un bar o en una fiesta y se liga a una mujer logrando que ésta tome sus llaves y lo lleve de regreso a su casa. Podría ser que el personaje logra un vínculo importante con uno de sus amigos al ver que éste está dispuesto a conducirlo a su casa. Quizá un amigo o un familiar recoge al personaje después de que éste ha tomado demasiado y, durante el trayecto a la casa, conectan a través de una conversación importante. En fin, las posibilidades son muchas, pero el reto es lograr que el hombre que pide apoyo no parezca débil. La gran falla de otras compañas a favor de conducir sin alcohol es que no han logrado mostrar que ceder las llaves es un acto verdaderamente valiente y admirable.

Además de que el personaje muestre cómo se pueden ceder las llaves, posteriormente, podría ser él quien le recomienda a los demás que hagan lo mismo. Al hacer dichas recomendaciones podría comunicar los riesgos de conducir bajo la influencia del alcohol, entre ellos la posible deportación.

Segundo mensaje prosocial para *Telenovela 2*:
Estrategias para sugerirle a los televidentes que consideren aplicar para una beca universitaria

Capítulos y fechas entre las cuales se presentarán los mensajes sobre las becas universitarias:

	Inicio del periodo	Fin del periodo
**Día**[5]	18 de abril, 2012	22 de mayo, 2012
**Capítulo**	61	85

Sobre la importancia de la educación universitaria:

Una educación universitaria abre muchas puertas. Hay estudios que demuestran que es más probable que las personas con título universitario logren encontrar el trabajo de sus sueños y obtengan un salario digno. Es más, las personas que cuentan con cuatro años de estudios universitarios ganan en promedio veinte mil dólares más que las personas que sólo cuentan con estudios de secundaria y preparatoria.

La educación superior también ayuda a que los estudiantes descubran nuevos horizontes. En la universidad se aprende sobre los grandes pensadores y sus descubrimientos. Es ahí donde hay una mayor probabilidad de tener contacto con la diversidad humana. Así es, los estudiantes universitarios tienen oportunidades de conocer a estudiantes y profesores que vienen de diferentes entornos.[6]

La falta de educación superior entre la comunidad latina/hispana:

La comunidad latina/hispana en los Estados Unidos ha crecido rápidamente en las últimas cinco décadas. El problema es que, a pesar de dicho crecimiento, la proporción de los jóvenes hispanos/latinos que aplican a las universidades ha sido limitada. También es cierto que son relativamente pocos los hispanos/latinos que, después de ser admitidos a una universidad, llegan a titularse.[7] La siguiente estadística lo dice todo: 30.5 por ciento de los adultos anglos cuentan con un título universitario, mientras que sólo el 12 por ciento de los adultos hispanos/latinos cuentan con dicho diploma.[8] Como lo muestra la siguiente gráfica, entre las

[5] El cálculo de las fechas está basado en que el programa se estrenará el 24 de enero.

[6] Para más información ver <http://yourwordstoday.org/about/college_is_important/>.

[7] Para más información ver <http://completionagenda.collegeboard.org/latino/>.

[8] Para más información ver <http://www.hsf.net/innerContent.aspx?id=32#challenge>.

diferentes etnias en los Estados Unidos, los hispanos/latinos viven en el rezago educativo.[9]

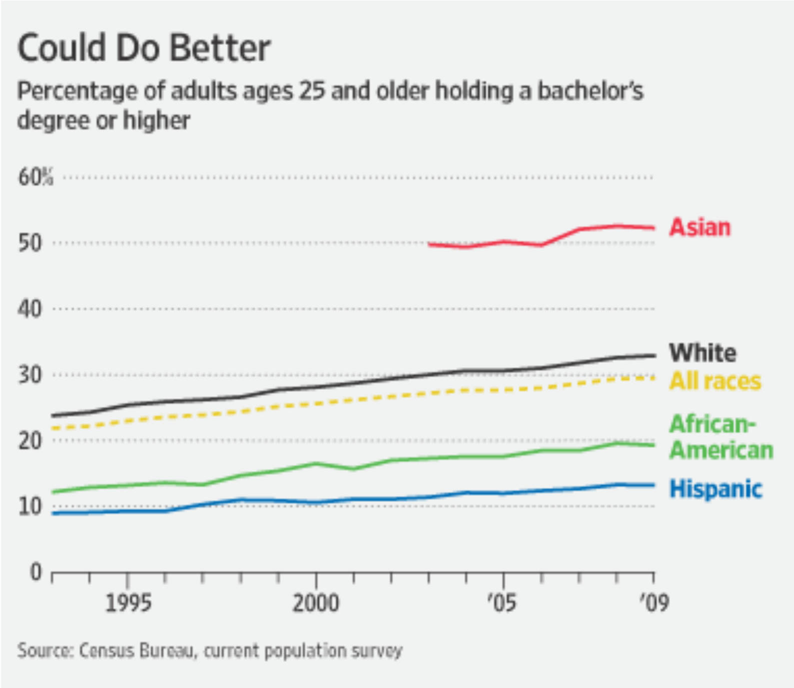

Los problemas antes descritos se deben a varios factores. Entre ellos está el hecho de que muchos de los hispanos/latinos no creen que podrían cubrir los gastos de una educación universitaria, pero la verdad es que hay más de $168 mil millones de dólares disponibles para ayudar a que las familias financien los sueños educativos de los hijos. Lo que falta es información.

El mensaje:

Dado todo lo anterior, se promoverá la página <www.tuspalabrasdehoy.org> a través de la novela. Dicha página depende de la Hispanic Scholarship Fund, una organización sin fines de lucro, y es una fuente de información muy completa sobre el sistema universitario estadounidense.

Es importante que el mensaje se presente varias veces y en diferentes capítulos para así tener un mayor impacto sobre la audiencia. Los miles de televidentes que estarán siguiendo el programa deben poder ver claramente cómo se ingresa la dirección de la página de Internet en el buscador.

---

9 Para más información ver <http://online.wsj.com/article/SB10001424052702304510704575562352066596296.html >.

Mensaje pro social para *Telenovela 3*:
Estrategias para sugerirle a los televidentes que se ejerciten y coman sanamente

Capítulos entre los cuales se deberá presentar el mensaje sobre el ejercicio y la alimentación sana:

	Inicio del periodo	Fin del periodo
**Capítulo**	76	85

El reto que enfrenta la población hispana:

- Uno de cada tres niños y dos de cada tres adultos en los Estados Unidos sufren de sobrepeso u obesidad.
- La población hispana es el segundo grupo social con mayor índice de obesidad en los Estados Unidos. Más del 30 por ciento de los hispanos enfrentan este problema.
- Como bien dijo Elena Rios, la Presidenta del National Hispanic Medical Association, "Los Latinos tienen el índice de obesidad más elevado en el país, esto de por si causará un aumento en los índices de diabetes y en la enfermedad del corazón en nuestra comunidad".

Dado todo lo anterior, el reto está en lograr que los televidentes se ejerciten y coman sanamente.

Información (y páginas de internet) sobre el ejercicio:

Los beneficios del ejercicio:

- Previene enfermedades
- Disminuye el riesgo de sufrir problemas cardiacos
- Evita la diabetes de tipo 2
- Reduce el sobrepeso
- Ayuda con los dolores musculares en la espalda
- Fortalece los huesos y evita el osteoporosis
- Ofrece bienestar psicológico

Información sobre la alimentación sana:

Los beneficios de comer sanamente:

1. Disminuye el sobrepeso
2. Mejora la calidad de vida
3. Reduce el riesgo de presentar problemas cardiacos, carencias en los huesos, diabetes de tipo 2, presión alta, y algunos casos de cáncer
4. Ayuda a evitar gastos médicos

Más información sobre los beneficios del ejercicio y la alimentación sana:

El tema de la salud es tan importante que la primera dama, Michelle Obama, se ha dedicado a promover la actividad física y el consumo de comida saludable. Su campaña lleva el nombre de "Let's Move!" y ofrece información resumida y en español a través del siguiente link.

Sugerimos los siguientes mensajes pro sociales:

Agradeceremos que el mensaje se repita tantas veces como se pueda entre los capítulos 36 y 60 para lograr el impacto deseado.
Ahora, hay varias maneras de presentar el mensaje. Así es, hay más de una opción de cómo sugerirle a los televidentes que se ejerciten y que coman sanamente. Por ejemplo, puede ser que varios de los personajes se interesen en bajar de peso, mejorar su salud, verse más sexy, etc. Quizá varios de los personajes estén teniendo problemas de salud o problemas en su vida romántica y busquen opciones de cómo mejorar su suerte. Dichos personaje podríans:

1. **Comprar verduras y frutas congeladas, mismas que pueden usarse para cocinar**
2. **Sustituir el consumo de leche normal por leche baja en grasa**
3. **Usar el aceite de oliva en vez de la mantequilla al cocinar**
4. **En vez de cocinar con huevos común y corrientes, utilizar sustitutos de huevo o únicamente las claras de huevo**
5. **Consumir sustitutos de sal y azúcar**
6. **Conseguir una membresía en el YMCA o el gimnasio más cercano, ya que la actividad física diaria es muy importante**

Para más información por favor no dude en contactarnos:

Paul Lagunes: 203-809-8642 o paul.lagunes@yale.edu (en español)

Elizabeth Paluck: 857-756-8933 (en ingles)

Mensaje pro social para *Telenovela 3*:
Estrategias para sugerirle a los televidentes que utilicen sillas infantiles para el auto

Capítulos entre los cuales se deberá presentar el mensaje sobre el uso de sillas infantiles para el auto:

	Inicio del periodo	Fin del periodo
**Capítulo**	86	110

El reto que enfrenta la población hispana:

- Los accidentes automovilísticos representan la principal causa de muerte de niños entre uno y doce años de edad.
- El riesgo de morir en un accidente automovilístico es dos veces mayor entre la juventud hispana que entre la juventud anglosajona. La razón es que los jóvenes hispanos suelen no usar cinturón de seguridad y los bebés hispanos suelen no estar sujetos a una silla infantil para el auto.
- Es importante tomar en cuenta que en absolutamente todos los estados de la unión norteamericana es una obligación legal sujetar a los bebés a una silla especial en el auto.
- El problema es que estas sillas para bebé suelen costar caras y suelen ser difíciles de utilizar. También es cierto que, según varios estudios, las mamás latinas no están acostumbradas a utilizar sillas infantiles para el auto. Muchas de ellas creen que no cargar a sus bebés en brazo demuestra falta de cariño.[10]

Dado lo anterior, sugerimos que se utilice una historia dramática y emocional para describir los problemas que representa esta realidad. Para promover la inversión en una silla infantil para el auto y el uso de dicho producto **la novela podría mostrar a un padre o a una madre que pierden a su bebé por estarlo(la) cargando en brazo dentro de un automóvil que se impacta.**

A continuación está la información clave que pedimos sea comunicada a los televidentes:

1. **Compra una silla infantil para el auto:** Visita una tienda, busca quien te atienda, y comunícale al empleado la edad de tu bebé para que te ayuden a encontrar la silla adecuada. ¡Hay varias opciones!

[10] Para más información ver: http://www.preventioninstitute.org/tools/focus-area-tools/communities-taking-action-profiles-of-health-equity/location/381.html

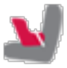

Se recomienda comprar una silla infantil para auto nueva. Es difícil saber si las sillas infantiles de segunda mano aun funcionan como deben.

2. **Una silla mirando hacia la parte trasera del auto:** Una silla infantil que mira hacia la parte trasera del auto es la opción idónea para los bebés. De sufrir un accidente, dicha silla ayuda a proteger el cuello y la espina dorsal del bebé.

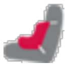

3. **Una silla mirando hacia la parte frontal del auto:** Una silla infantil que mira hacia la parte frontal del auto también tiene sus ventajas. Dicha silla sostiene al bebé en la eventualidad de un accidente automovilístico.

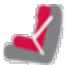

4. **Una silla "de apoyo":** Una silla "de apoyo" eleva al niño de tal forma que el cinturón del coche sirve como buen sostén. En caso de utilizar una silla de este tipo, es importante verificar que la parte inferior del cinturón pase sobre la pantorrilla del niño y la parte superior pase por el hombro y el pecho del mismo. El cinturón jamás debe descansar sobre el estómago o el cuello.

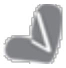

5. **Por último, es importante que TODOS utilicen el cinturón de seguridad.**

Información adicional:

1. Es importante recibir asesoría para verificar que estás utilizando tu silla infantil para auto de forma adecuada.
2. Métodos para utilizar una silla infantil para el auto: http://www.safercar.gov/parents/RightSeat.htm
3. Para saber si una silla infantil para el auto está siendo usada de forma adecuada: http://www.nhtsa.gov/cps/cpsfitting/index.cfm
