# Supplementary material for: Does Product Placement Change Television Viewers’ Social Behavior?
Source: PLoS One. 2015 Sep 23;10(9):e0138610. doi: 10.1371/journal.pone.0138610 (PMC4580471; doi:10.1371/journal.pone.0138610)
Supplement: S1 Link — Web version of the figures. (DOCX) [file pone.0138610.s011.docx]

1) Link to Paluck et al. replication code and data:

<https://osf.io/q7rsm/>

2) Link to web version of the figures:

<http://bit.ly/1Ldijew>
